# Potential health impacts and costs of active case finding guided by *Mycobacterium tuberculosis* immunoreactivity survey results in Blantyre, Malawi: A mathematical modeling study

**DOI:** 10.1371/journal.pmed.1005272

**Published:** 2026-10-07

**Authors:** Sun Kim, Melike Hazal Can, Hannah M. Rickman, Mphatso D. Phiri, Marriott Nliwasa, Tisungane E. Mwenyenkulu, Kuzani Mbendera, Stéphane Verguet, Marcia C. Castro, Elizabeth L. Corbett, Peter MacPherson, Ted Cohen, Nicolas A. Menzies

**Affiliations:** 1 Department of Global Health and Population, Harvard T.H. Chan School of Public Health, Boston, Massachusetts, United States of America; 2 Clinical Research Department, London School of Hygiene & Tropical Medicine, London, United Kingdom; 3 Malawi Liverpool Wellcome Programme, Blantyre, Malawi; 4 Department of Clinical Sciences, Liverpool School of Tropical Medicine, Liverpool, United Kingdom; 5 Helse Nord Tuberculosis Initiative, Department of Pathology, Kamuzu University of Health Sciences, Blantyre, Malawi; 6 National Tuberculosis and Leprosy Elimination Program, Ministry of Health, Lilongwe, Malawi; 7 School of Health & Wellbeing, University of Glasgow, Glasgow, United Kingdom; 8 Department of Epidemiology of Microbial Diseases, Yale School of Public Health, New Haven, Connecticut, United States of America; University of Ottawa, CANADA

## Abstract

**Background:**

Active case finding (ACF) for tuberculosis (TB) can reduce transmission, yet efficient targeting requires high-quality surveillance data. We investigated the potential costs and impact of targeted ACF, guided by local estimates of the annual risk of TB infection (ARTI) derived from *Mycobacterium tuberculosis* (*Mtb*) immunoreactivity survey data in children aged <5 years.

**Methods and findings:**

Using mathematical models parameterized with local data, we compared three case-finding approaches across 33 urban neighborhoods in Blantyre, Malawi: passive case finding (PCF) only; PCF with untargeted ACF; and PCF with ARTI-guided targeted ACF. Health outcomes (life expectancy, disability-adjusted life years [DALYs]) and costs were estimated using a Markov microsimulation model. Costs were assessed from health system and societal perspectives. Results were calculated for different assumptions about the relationship between ARTI and true TB prevalence.

Compared to PCF-only, untargeted ACF was estimated to improve life expectancy by 3.3 years (95% credible interval (CrI) [1.7, 5.4]) for individuals with TB disease, but at high cost. Targeted ACF covering half of the study population was estimated to identify 80% of all individuals with TB and achieved a lower cost per DALY averted (US$400, 95% CrI [20,1,000]) than untargeted ACF (US$700, 95% CrI [100, 1,500]). In the main analysis, these cost-effectiveness ratios exceeded available cost-effectiveness thresholds for Malawi, although cost-effectiveness improved under assumptions of higher TB prevalence or greater transmission reduction. The findings are based on mathematical modeling and are subject to several limitations, including assumptions regarding the relationship between ARTI and TB prevalence, which remains uncertain.

**Conclusions:**

Targeting ACF using *Mtb* immunoreactivity survey data could substantially improve health impact and cost-effectiveness compared to untargeted approaches. However, low-cost approaches for collecting these data are needed for wider adoption.

## Author summary

**Why was this study done?:**

- Active case finding (ACF) can reduce tuberculosis (TB) transmission, but implementing ACF across entire populations is resource-intensive.
- TB programs need practical ways to identify locations where targeted ACF will have the greatest impact.
- We investigated whether local estimates of the annual risk of TB infection (ARTI) obtained from *Mycobacterium tuberculosis (Mtb)* immunoreactivity surveys in young children could be used to guide geographic targeting of ACF.

**What did the researchers do and find?:**

- We developed a mathematical modeling framework to compare passive case finding, untargeted ACF, and ARTI-guided targeted ACF across 33 neighborhoods in Blantyre, Malawi.
- Targeting communities with higher estimated ARTI for ACF identified a larger proportion of TB cases than untargeted ACF at the same population coverage.
- Targeted ACF produced greater health gains and lower costs per disability-adjusted life year (DALY) averted than untargeted ACF, although both approaches remained above commonly cited cost-effectiveness thresholds for Malawi.
- Cost-effectiveness improved in sensitivity analyses assuming higher initial TB prevalence or greater impact on secondary transmission.

**What do these findings mean?:**

- Geographic targeting using Mtb immunoreactivity survey data could substantially improve the efficiency of community TB case-finding programs.
- These findings support further evaluation of immunoreactivity surveys as a surveillance tool for prioritizing TB interventions in high-burden settings.
- Our findings are based on mathematical modeling and are subject to several limitations, including assumptions regarding the relationship between ARTI and TB prevalence, transmission dynamics, and intervention effectiveness. Further empirical and implementation studies are needed to better characterize these relationships and evaluate the effectiveness of ARTI-guided targeted active case finding in real-world settings.

## Abstract 3

In this modelling study, Sun Kim and colleagues explore whether tuberculosis active case finding can be more efficiently targeted using local Mycobacterium tuberculosis immunoreactivity survey data from children in Blantyre, Malawi. Their findings suggest that targeting screening efforts to higher-risk areas may identify a large proportion of TB cases and offer better cost-effectiveness than untargeted approaches.

## Introduction

Tuberculosis (TB) remains a major global public health threat, especially in regions with a high burden of HIV [1]. Active case finding (ACF) is recommended by the World Health Organization to identify individuals with untreated TB, and involves offering systematic screening to community members regardless of symptoms. ACF can be effective in reducing the prevalence of undiagnosed infectious TB, and therefore interrupting community transmission [2]. While it holds promise, untargeted implementation across entire populations may be expensive and inefficient. Targeting ACF to high-prevalence areas may increase efficiency; however, data to identify such areas (e.g., subnational TB prevalence surveys) are usually not available due to the cost and large sample sizes required to conduct such surveys [3]. TB case notification data could be used as a proxy for prevalence to guide ACF targeting, but areas with high notification rates may reflect capacity for prompt care-seeking behavior and better access to health services, rather than underlying disease burden. New approaches to prioritizing communities with a high burden of undiagnosed disease are required, especially in the context of declining TB burdens [4].

Malawi is a low-income country in southeastern Africa, with TB incidence of 119 per 100,000 and nearly half of all cases occurring among persons with HIV [1]. In Blantyre, the second most populous city in the country, high TB notification rates are frequently observed in better-resourced neighborhoods with stronger health infrastructure, potentially obscuring areas with high levels of undiagnosed TB [4,5]. Like many high TB burden countries, Malawi’s National Tuberculosis and Leprosy Elimination Program (NTLEP) undertakes ACF, including community-based door-to-door outreach and mobile van-based diagnostic units equipped with Xpert and digital chest X-ray [6]. But determining which communities should be prioritized for ACF is challenging.

Recent findings from a *Mycobacterium tuberculosis* (*Mtb*) immunoreactivity survey using convenience samples of healthy children under five years of age attending primary care in Blantyre offer an alternative surveillance approach for targeting ACF [7,8]. The survey measured *Mtb* immune response using interferon-gamma release assays (IGRAs), providing neighborhood-level estimates of the annual risk of TB infection (ARTI) [7,9–11]. ARTI estimates in young children may be particularly informative for identifying areas of recent transmission and elevated force of infection [12–14], and thus useful for prioritizing locations where undiagnosed TB disease is more likely to be found.

In the present study, we used mathematical modeling to assess the potential health and economic impact of ARTI-guided targeted ACF in Blantyre to inform how surveillance innovations might contribute to more efficient and impactful TB prevention efforts.

## Methods

### Study setting and population

Blantyre is Malawi’s second-largest city and the country’s most densely populated urban center, with an estimated population of 800,000 [15]. Adult HIV prevalence is estimated to be 14%, among the highest recorded nationally [16]. In 2019, the prevalence of TB among adults was estimated at 215 per 100,000, based on a prevalence survey conducted as part of a cluster-randomized trial [17,18]. A large proportion of Blantyre’s population resides in informal settlements on underdeveloped land, with limited access to healthcare and other municipal services, despite TB and HIV care being free at the point of delivery [17,19].

The study focused on 33 urban neighborhoods (total estimated population of 266,710), defined by community health worker catchment areas and selected based on population density and proximity to 3 study clinics [20]. Recent neighborhood ARTI estimates were derived from a *Mtb* immunoreactivity survey, with methods described in detail by Rickman and colleagues (2025) [7]. These ARTI estimates were used to create scenarios for targeted ACF campaigns, as described below.

### Intervention scenarios

In collaboration with representatives from Malawi’s NTLEP, we defined three hypothetical intervention scenarios. The first scenario, (‘*passive case finding [PCF] only*’), served as a counterfactual baseline with no ACF, in which individuals with TB were only diagnosed if they self-presented to a health facility. The second scenario (*‘untargeted ACF’*) involved PCF in combination with untargeted ACF, implemented uniformly across all clusters regardless of underlying epidemiological differences. The third scenario (*‘targeted ACF’*) involved PCF in combination with targeted ACF, where clusters were prioritized sequentially from highest to lowest ARTI, as estimated from *Mtb* immunoreactivity survey data.

In both ACF scenarios, mobile diagnostic units equipped with digital chest X-ray and Xpert MTB/RIF were deployed to communities, accompanied by community health workers. Community members were invited for screening, beginning with a standardized symptom questionnaire. Individuals reporting TB symptoms (prolonged cough), or with any abnormality on chest X-ray, were asked to provide sputum for testing with Xpert MTB-RIF. Individuals with Xpert-confirmed TB were linked to treatment services. For both ACF scenarios, we calculated results for a one-time community-wide ACF intervention based on a previous randomized trial in Blantyre [20]. PCF was assumed to occur through the routine health system, with symptomatic clinic attendees receiving clinical evaluation and bacteriological testing through the Malawi Ministry of Health primary and secondary healthcare systems. As compared to ACF, individuals identified through PCF were assumed to face additional risks of mortality before and during treatment, and elevated rates of post-TB sequelae following treatment completion. Fig 1 presents a schematic of the diagnostic pathways and outcomes under each scenario.

**Fig 1:**
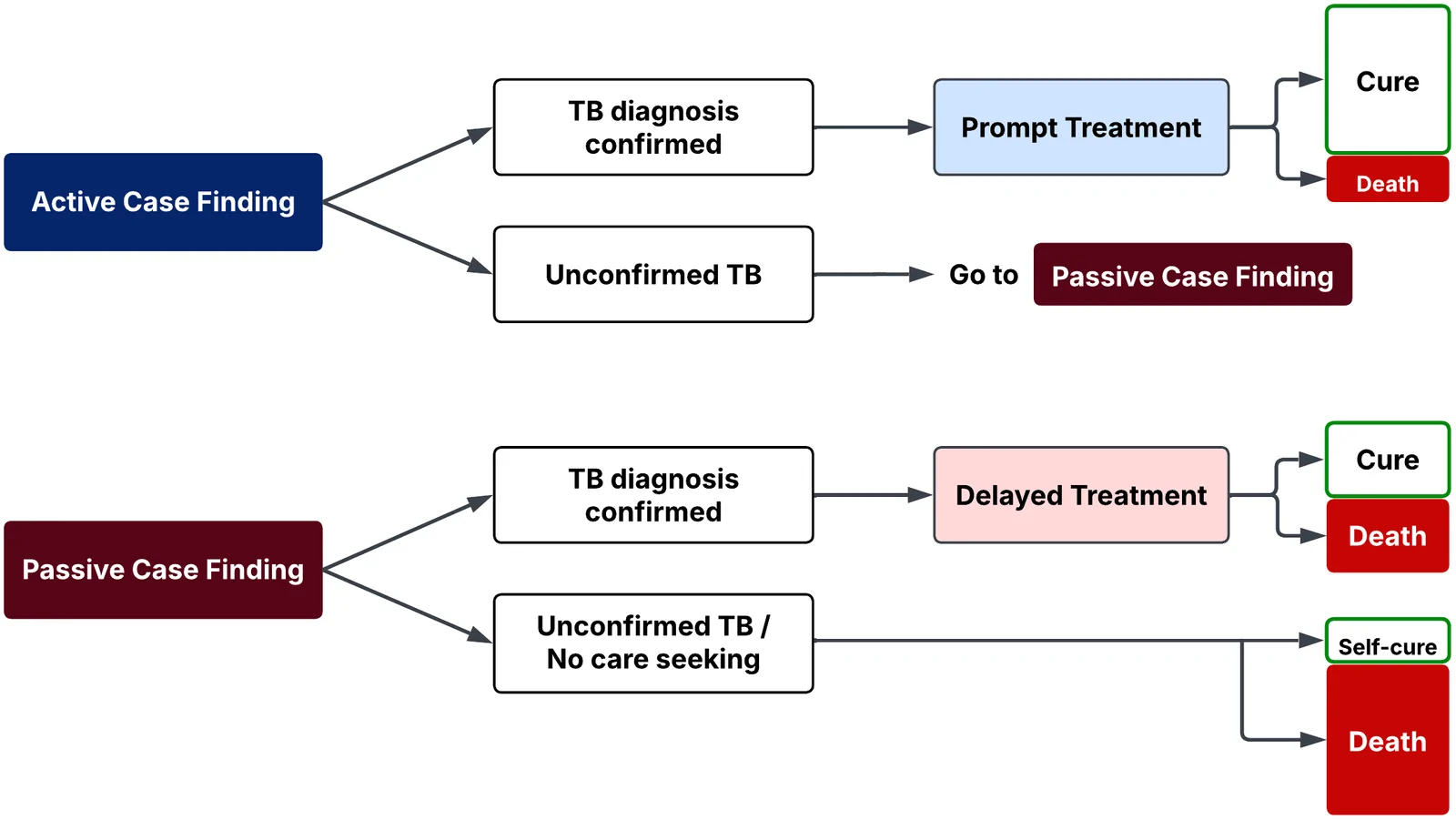
Pathways and outcomes for individuals with TB disease under active and passive case finding. The size of each outcome box (cure or death) is illustrative, and reflect relative frequency under each scenario, with larger boxes indicating more common outcomes and smaller boxes indicating less common outcomes.

### Health and cost outcomes

Health outcomes included life expectancy, TB-attributable mortality, and disability-adjusted life years (DALYs). DALYs were calculated as the sum of years of life lost (YLLs) and years lived with disability (YLDs) attributable to TB or HIV. Cost outcomes included both health system expenditures as well as patient-incurred costs, and were expressed in 2024 dollars (USD). Since TB services are provided free of charge in Malawi, patients were assumed to face no direct medical costs. Direct non-medical costs included transport, food, and accommodation during healthcare visits. Indirect costs reflected income loss or the value of time lost by patients and their households during the TB episode [21]. The incremental health benefits of ACF were calculated based on the consequences for individuals receiving ACF (benefits for individuals receiving true-positive diagnoses and harms for individuals receiving false-positive diagnoses) as well as health benefits resulting from averted secondary TB cases. Incremental costs for ACF scenarios were calculated from the direct costs of the ACF intervention (assumed to cost US$7 per person receiving ACF) and associated TB treatment, cost-savings from averted TB diagnosis and treatment through passive diagnosis, cost-savings resulting from averted secondary cases, and changes in ART costs due to changes in survival among individuals with HIV. We calculated all costs and health outcomes over the lifetime of affected individuals. For the calculation of incremental cost-effectiveness ratios (ICERs), costs and health benefits (DALYs averted) were both discounted at a rate of 3% per year.

### Modeling approach for individuals receiving ACF or PCF

We used a published Markov simulation model [22] to estimate lifetime health outcomes and TB care costs for individuals with or without TB and/or HIV under each intervention scenario. Using this model a cohort of 10,000 individuals representing the ACF target population was exposed to different TB diagnosis and treatment scenarios and tracked over time to estimate cumulative health outcomes and costs. Individuals were represented in the model by starting age (mean: 34.0, IQR: 26.0–40.0), sex (75.0% male for individuals with TB), HIV and ART status (13.6% HIV–positive, 70.1% of these on ART), CD4 cell count for individuals with HIV (mean: 274 cells/μL, IQR: 68–372 cells/μL), and FEV1 percentage as a measure of TB-associated lung damage (mean: 63%, IQR: 52%–75% for individuals with prevalent TB). Fig A in S1 Appendix provides a schematic of the model structure. In the model, individual survival and health status were updated with a weekly timestep, until each individual in the simulated cohort was dead. HIV-associated immune function was tracked through changes in CD4 cell count, which declined for individuals not receiving ART, and rebounded among individuals receiving ART. Individuals with HIV had greater HIV-associated mortality and disability weights, determined by CD4 cell count and ART receipt. HIV-associated immune function also affected rates of TB progression and probability of self-cure. For individuals with untreated TB, those receiving ART were assumed to have higher rates of diagnostic evaluation compared to individuals not receiving ART. Lung function was tracked through changes in FEV1 percentage, which was assumed to decline for individuals with untreated TB disease, rebound during TB treatment, and continue to improve following successful TB treatment. The extent of lung function recovery following TB cure was determined by the minimum FEV1 value reached before treatment initiation. FEV1 percentage was assumed to continue to decline for individuals receiving a false-negative TB diagnosis, lost to follow-up from treatment before TB cure, or with ongoing disease following treatment completion. TB-associated mortality and disability were determined by TB status and FEV1 percentage. Individuals surviving TB disease were assumed to face ongoing morbidity and mortality risks due to post-TB sequelae. The magnitude of these risks was based on the extent of residual long impairment as measured by FEV1 percentage. As compared to the earlier application of the model [22], this analysis adopted higher values for the FEV1 distribution for individuals with TB, reflecting a milder distribution of TB disease compared to individuals detected through passive case detection. Key model parameters are provided in Table A in S1 Appendix, and the model was programmed in R and C++. Additional details are reported in Can and colleagues, 2025 [22]. Modeled individuals were stratified by TB and HIV status and were simulated under the diagnostic pathways defined in each scenario.

Model inputs were drawn from multiple sources, including cluster-level ARTI estimates [7], a TB case notification dataset (2022) with residential geolocation collected via tablet-based electronic entry [4,23,24], Blantyre TB prevalence survey data (2019) [25], and population estimates from World Pop (2020) [15]. Cost inputs were based on published literature and NTLEP reports, and included unit costs for the ACF intervention, TB diagnosis, treatment, and HIV-related care, as well as patient-incurred costs (Table B in S1 Appendix).

### Approach for estimating secondary TB cases averted by ACF

We estimated the number of secondary TB cases averted based on the numbers of individuals with TB diagnosed and treated through ACF and the reproductive number for Blantyre. The reproductive number was estimated from the secular trend in TB incidence (assumed to be proportional to the TB notification rate), and published estimates of the generation time [26–28]. For individuals with TB identified through ACF we assumed transmission would be reduced by 50% (with an uncertainty interval from 25% to 75%) as compared to the PCF scenario. This was based on the rationale that a single cross-sectional ACF round will identify individuals approximately halfway through their infectious period, on expectation. We used the reproductive number to calculate the geometric series of future TB cases averted by ACF. For each averted TB case, incremental health benefits and costs were based on the health and economic outcomes for individuals with versus without TB, calculated using the Markov model. We considered costs and health benefits associated with all future secondary TB cases averted by ACF. In sensitivity analyses, we recalculated costs and health consequences i) assuming ACF would reduce transmission among detected TB cases by 75% (vs. 50% in the main analysis), ii) using a higher TB prevalence estimate (452 per 100,000, based on data for Blantyre extracted from Malawi’s 2013–2014 national TB prevalence survey [29], versus 215 per 100,000 in the main analysis), and iii) excluding transmission benefits entirely.

### ACF algorithm performance

Based on input from the Malawi NTLEP and implementing partners, we assumed that ACF would be undertaken with an algorithm in which individuals with any reported cough would be tested with Xpert. Individuals not reporting a cough would receive chest X-ray, and those with any abnormality on this X-ray would also receive Xpert. Individuals testing Xpert positive would be diagnosed with TB. Under the ACF scenarios, we assumed that all residents in a given cluster would be offered ACF, 10.2% of those offered ACF would refuse or be unable to provide sputum [30], 5.0% of those diagnosed with TB would be lost to follow-up before treatment initiation (based on NTLEP input), and individuals initiating treatment would experience treatment outcomes (death, loss to follow-up, failure, and cure) consistent with reported treatment outcomes data [31].

ACF algorithm sensitivity was calculated as (1 − (1 − Sens_symp_)*(1 − Sens_cxr_))*Sens_xpert_, where Sens_symp_, Sens_cxr_, and Sens_xpert_ represent the sensitivity of the symptom screen, chest X-ray, and Xpert, respectively. Algorithm specificity was calculated as 1 − (1 − Spec_symp_*Spec_cxr_)*(1 − Spec_xpert_), where Spec_symp_, Spec_cxr_, and Spec_xpert_ represent the specificity of the symptom screen, chest X-ray, and Xpert, respectively. Values and sources for these calculations are shown in Table C in S1 Appendix.

### Statistical analysis

Simulation outputs were aggregated at the cluster level to estimate TB disease detection, life expectancy, DALYs, and costs under each intervention scenario. These results were used to calculate incremental differences in health outcomes and costs compared to the *PCF-only* scenario. ICERs comparing ACF scenarios to *PCF-only* were computed at both the cluster level and across all clusters combined. The analysis was conducted over a lifetime horizon.

To evaluate the efficiency of ARTI-guided targeted ACF, we assumed that cluster TB prevalence was positively associated with ARTI. We operationalized this by assuming that the TB prevalence in cluster i ($\pi_{i}$), is equal to $k\cdot A_{i}^{P}$, where $A_{i}$ is ARTI in cluster i, P parameterizes the strength of relationship between ARTI and TB prevalence, and k is a normalizing constant calculated to ensure average prevalence across clusters equals 215 per 100,000:$\pi_{i}=k\cdot A_{i}^{P}$.

We varied the value of *P* from 0.0 (no relationship between TB prevalence and ARTI) to 1.0 (TB prevalence linearly proportional to ARTI), in increments of 0.2. The number of individuals with prevalent TB in each cluster was calculated as $\pi_{i}\cdot N_{i}$, where $N_{i}$ is cluster population.

Using these cluster-specific TB prevalence estimates, we then computed the cumulative number of TB cases identified, DALYs averted, and incremental costs as clusters were sequentially added to the ARTI-guided targeted ACF intervention in order of descending ARTI. To further assess the implications of geographic targeting, we modeled a scenario in which ACF was implemented in clusters comprising half of the total population, starting with clusters with the highest ARTI. Clusters were ranked and included sequentially until reaching 50% population coverage. We compared this targeted approach to an untargeted strategy in which the same population coverage was achieved through random selection of clusters.

We computed 95% credible intervals as the 2.5th and 97.5th percentiles of the sample of estimates for each outcome, as generated from 1,000 randomly drawn parameter sets, reflecting uncertainty in input parameters. All analyses were performed in R software (v4.4.3), using the Rcpp package [32]. ChatGPT (OpenAI; GPT-5.5) was used solely to support grammar and language editing and to assist with limited code development and debugging. All Artificial Intelligence (AI)-assisted text was checked and revised by the authors. All AI-assisted code was reviewed, tested, and validated against the intended model specifications and outputs. The authors retain full responsibility for the study, code, and final manuscript.

### Ethics statement

This mathematical modeling study used previously collected, published, and anonymized data and involved no new participant recruitment or data collection. The data were fully anonymized and posed no risk of individual identification; additional ethical approval and informed consent were therefore not required.

### Inclusivity in global research

Additional information regarding the ethical, cultural, and scientific considerations specific to inclusivity in global research is included in the Supporting Information (S2 Appendix).

## Results

### Baseline characteristics of clusters

Fig 2 presents the geographic distribution of clusters included in the ARTI survey. The 33 urban clusters included in the analysis demonstrated substantial variation in population size, TB burden, and HIV prevalence. Estimates of cluster-specific ARTI ranged from 0% to 9% (Table D in S1 Appendix).

**Fig 2:**
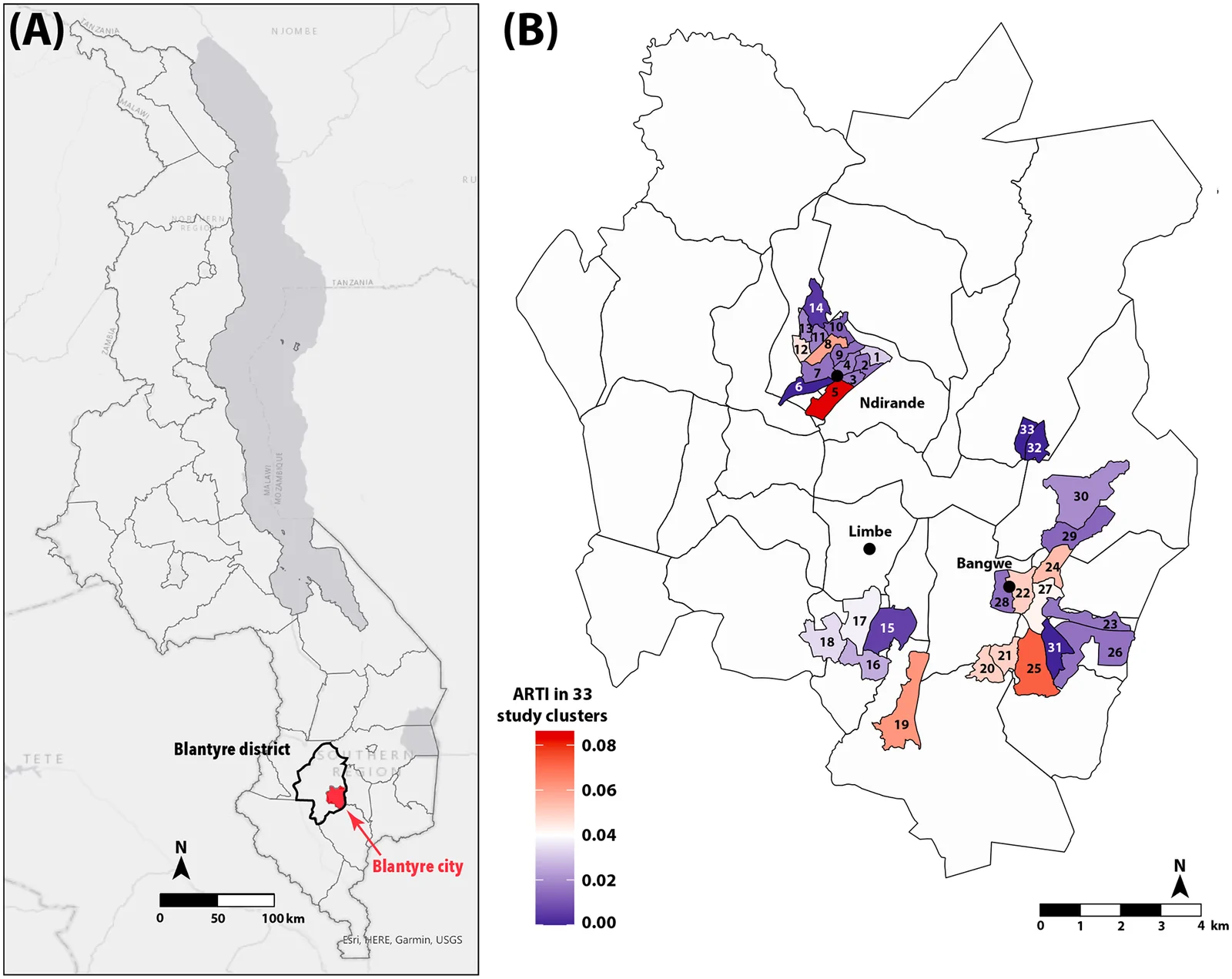
Map of Malawi (Panel A) and Annual Risk of TB Infection (ARTI) by cluster in Blantyre city (Panel B). Base map sources: **(A)** Esri World Light Gray Canvas (Esri, HERE, Garmin, USGS), accessed via ArcGIS Online, and Humanitarian Data Exchange (CC BY-IGO), https://data.humdata.org/dataset/cod-ab-mwi and **(B)** mlwdata GitHub repository (MIT License; Copyright 2019 Peter MacPherson), https://github.com/petermacp/mlwdata.

### Case detection under varying ARTI predictive power

The number of TB cases identified under the ACF scenarios varied depending on the assumed predictive power of ARTI. Fig 3 shows the cumulative number of individuals with TB detected as clusters were sequentially added to the targeted ACF intervention, under predictive power assumptions ranging from *P* = 0 to *P* = 1. When ARTI was assumed to be highly predictive of TB prevalence, a greater proportion of cases was identified in the earliest-added clusters, resulting in a sharply increasing cumulative curve that reflects concentrated yield in a small number of high-ARTI clusters. As predictive power decreased, the distribution of cases became more uniform across clusters, though still yielding greater case detection for targeted ACF compared to untargeted ACF.

**Fig 3:**
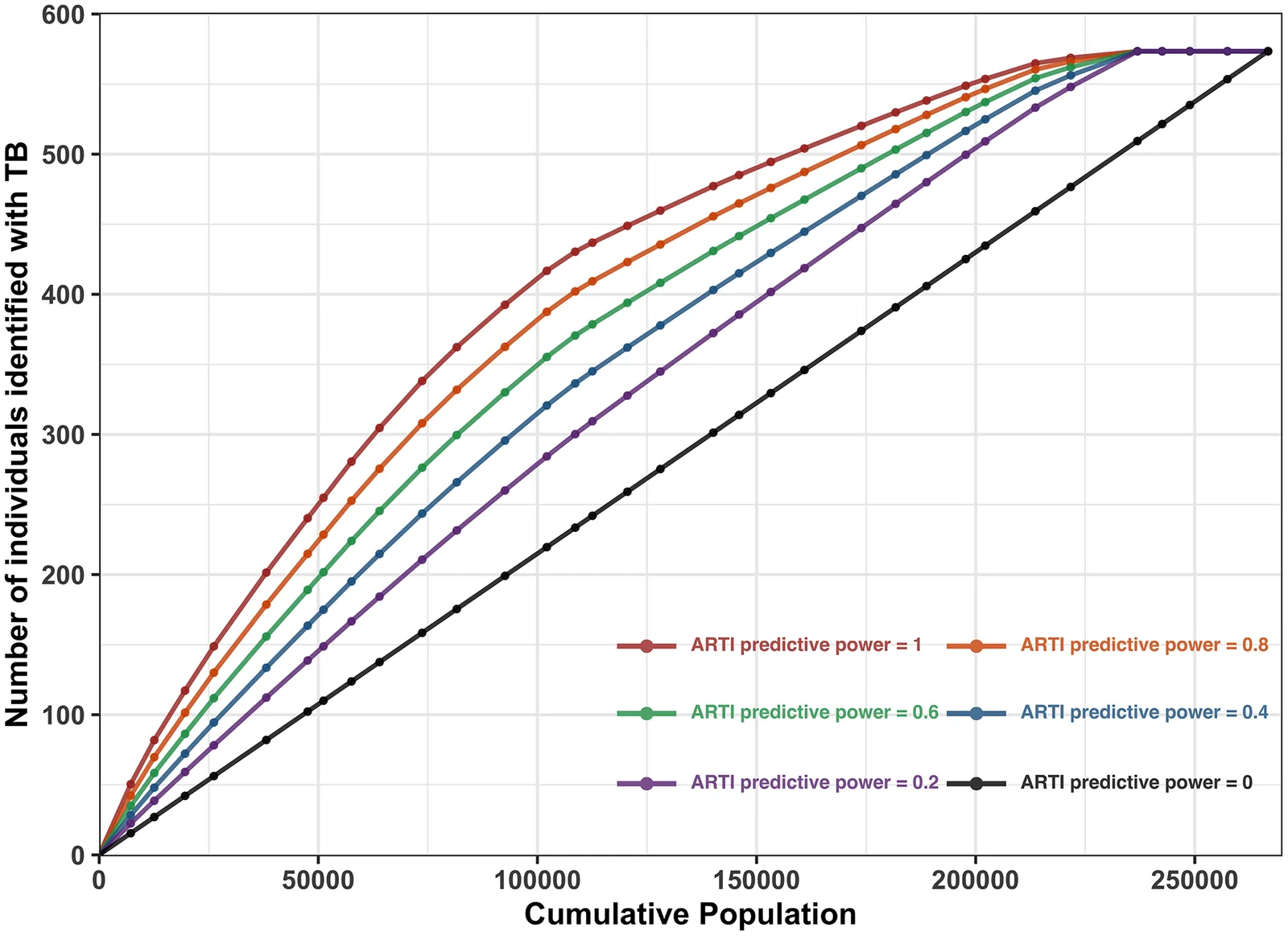
Number of individuals with TB disease identified through active case finding (ACF) based on annual risk of TB infection (ARTI) predictive power. Excluding participants with false-positive results.

### Health impact for individuals receiving ACF

Under both ACF scenarios, ACF implementation resulted in improved health outcomes for individuals with TB. For HIV–negative individuals with TB, ACF increased life expectancy by an average of 3.3 years (95% credible interval (CrI) [1.7, 5.4]) compared to PCF alone, while HIV–positive individuals gained an average of 2.1 years (95% CrI, [0.8, 3.7]) with ACF (Table 1). False-positive results among individuals without TB, leading to unnecessary TB treatment, were associated with a small decrease in life expectancy of 3.2e^−5^ years (95% CrI [3.9e^−6^, 7.0e^−5^]) among HIV–negative individuals and 1.4e^−5^ years (95% CrI [0, 4.3e^−5^]) among people living with HIV.

**Table 1:** Estimated life expectancies (LE) and Disability Adjusted Life Years (DALYs) under ACF and PCF scenarios.

Category		Under ACF scenario	Under PCF scenario	Incremental Difference
**Life Expectancy from TB and HIV**				
Individuals with TB	HIV–negative	31.4 (29.3, 33.4)	28.1 (24.2, 31.5)	3.3 (1.7, 5.4)
Individuals with TB	HIV–positive	12.5 (6.5, 16.1)	10.4 (5.3, 14.0)	2.1 (0.8, 3.7)
Individuals without TB	HIV–negative	37.0 (36.7, 37.3)	37.0 (36.7, 37.3)	−3.2e^−5^ (−7.0e^−5^, −3.9e^−6^)
Individuals without TB	HIV–positive	15.7 (8.0, 20.2)	15.7 (8.0, 20.2)	−1.4e^−5^ (−4.3e^−5^, 0.0)
**DALYs from TB and HIV**				
Individuals with TB	HIV–negative	10.2 (6.8, 14.1)	16.0 (10.2, 23.0)	−5.8 (−9.3, −3.1)
Individuals with TB	HIV–positive	36.0 (31.3, 43.2)	38.8 (34.2, 44.7)	−2.8 (−5.0, −1.0)
Individuals without TB	HIV–negative	5.2e^−5^ (1.2e^−5^, 1.1e^−4^)	0.0 (0.0, 0.0)	5.2e^−5^ (1.2e^−5^, 1.1e^−4^)
Individuals without TB	HIV–positive	31.7 (25.3, 41.3)	31.7 (25.3, 41.3)	2.1e^−5^ (5.2e^−6^, 5.5e^−5^)

*DALYs disaggregated by YLDs and YLLs are available in Tables E and F in S1 Appendix. DALYs shown represent health losses to TB and or HIV.

ACF also led to substantial reductions in DALYs among individuals with TB. Among those who were HIV–negative, the average DALYs averted per person was 5.8 (95% CrI, [3.1, 9.3]), and among HIV–positive individuals, 2.8 DALYs were averted (95% CrI [1.0, 5.0]) (Table 1). The majority of these gains were driven by reductions in years of life lost (YLLs) (Tables E and F in S1 Appendix). Reductions in the share of deaths attributable to TB were also notable: under ACF, the proportion of TB-attributable deaths was 7.2 percentage points lower (95% CrI [3.3, 13.4]) among HIV–negative individuals and 14.2 percentage points lower (95% CrI [7.3, 23.6]) among HIV–positive individuals, compared with PCF (Table G in S1 Appendix).

### Secondary TB cases averted by ACF

With ACF, the reduction in transmission resulting from earlier TB identification and treatment through ACF was estimated to avert 0.88 (95% CrI [0.24, 2.29]) downstream TB cases per index case. Accounting for the estimated number of secondary cases averted and the health burden associated with each case, each TB case identified through ACF was estimated to yield an additional 5.08 (95% CrI [1.32, 15.04]) life-years gained and 8.98 (95% CrI [2.28, 26.83]) DALYs averted through reduced transmission.

### Cost-effectiveness of ACF by cluster

Cost-effectiveness ratios (comparing ACF to passive detection) varied substantially across clusters, driven by differences in HIV prevalence and estimated TB burden. Under the assumption of perfect predictive power (*P* = 1), the majority of clusters had ICERs below $1,000 per DALY averted, with a median of $845 (Table 2). As anticipated, the ACF intervention was dominated for clusters with zero ARTI, meaning that for these clusters (with assumed zero TB prevalence) ACF was estimated to incur higher costs and harm health outcomes.

**Table 2:** Cost-effectiveness of targeted active case finding (ACF) vs. no ACF by cluster, ordered by ARTI^1^.

Cluster No.	Population	ARTI (%)	Incremental cost (2024 USD)	DALYs averted	ICER^2^ (USD per DALY averted)
5	7,198	8.6	39,615	284	139
25	5,369	7.2	29,762	178	167
19	7,027	6.2	49,660	193	258
8	6,573	5.9	46,075	172	267
24	11,967	5.4	73,153	296	247
22	9,435	5.1	60,709	216	281
20	3,606	5.0	22,017	82	268
21	6,390	5.0	42,998	142	302
12	6,417	4.6	37,924	136	278
27	9,715	4.3	62,813	187	336
17	7,904	3.8	52,904	133	397
18	11,002	3.4	66,065	173	381
1	9,537	3.1	59,139	138	428
16	6,476	2.6	39,497	79	501
30	3,934	2.0	26,840	36	754
13	7,982	1.9	55,365	65	845
4	7,524	1.8	49,696	61	810
11	12,070	1.8	83,769	95	880
3	5,897	1.7	41,228	43	961
23	7,233	1.6	48,224	53	918
2	7,675	1.5	54,336	52	1,055
26	12,976	1.5	88,235	89	987
7	7,837	1.5	52,925	54	988
28	7,038	1.5	49,002	46	1,065
9	8,946	1.5	59,851	59	1,011
29	4,463	1.3	29,332	28	1,063
10	11,433	1.2	79,854	60	1,325
15	8,054	0.6	54,857	22	2,486
14	15,231	0.4	104,458	26	3,982
6	5,643	0.0	39,110	−0.15	Dominated^3^
31	6,323	0.0	43,817	−0.17	Dominated
32	8,574	0.0	59,422	−0.23	Dominated
33	9,263	0.0	64,193	−0.25	Dominated
Total^4^	266,710	–	1,766,845	3,199	677

^1^Results presented assume an ARTI (Annual Risk of TB Infection) predictive power of 1. See Tables H–L in S1 Appendix for results under alternative predictive power assumptions.

^2^ICER: Incremental Cost-Effectiveness Ratio. For the cost-effectiveness analysis, both DALYs and costs are discounted at a rate of 3% per year.

^3^Strategies labeled "Dominated" are more costly and less effective than PCF only.

^4^The total row reflects the impact of 100% ACF coverage across all 33 clusters.

Fig B in S1 Appendix shows how the total costs and health outcomes of ARTI-guided targeted ACF increased as more clusters were included in the intervention. Under perfect predictive power (*P* = 1), health benefits increased rapidly with initial investments, while as predictive power decreased, the returns to investment diminished, and more clusters were required to achieve the same level of health benefits.

### Comparison of targeted versus untargeted ACF

As shown in Table 3, a targeted ACF intervention including half of the total population consistently identified clusters with higher average TB prevalence, ranging from 362 to 269 per 100,000 as predictive power decreased from *P* = 1 to *P* = 0.2. This targeting approach captured a larger share of the total TB burden than untargeted ACF. For example, when ARTI was assumed to be perfectly predictive (*P* = 1), targeting half of the total population through ACF using prioritized clusters identified 81.5% of all prevalent TB cases, compared to only 50% in the untargeted scenario. As predictive power decreased from 1 to 0.2, ICERs increased from $400 (95% CrI [20, 1,000]) to $500 (95% CrI [60, 1,200]) per DALY averted. In contrast, untargeted ACF (*P* = 0) produced an ICER of $700 (95% CrI [100, 1,500]) per DALY averted.

**Table 3:** Cost-effectiveness of targeted active case finding (ACF) vs. untargeted ACF at 50% cluster coverage^1^.

	Average TB prevalence among selected clusters (per 100k)	Fraction of total cases identified (%)	Incremental cost^2^ in thousand	DALYs averted	ICER compared to no ACF
**Targeted** **(*P* = 1)**	362	81.5	859 (91, 1,555)	2,613 (1,196, 6,104)	411 (20, 979)
**Targeted** **(*P* = 0.8)**	342	77.5	862 (124, 1,546)	2,484 (1,137, 5,804)	433 (26, 1,029)
**Targeted** **(*P* = 0.6)**	320	72.9	866 (158, 1,538)	2,338 (1,070, 5,462)	460 (34, 1,085)
**Targeted** **(*P* = 0.4)**	295	67.8	870 (197, 1,529)	2,174 (995, 5,080)	495 (44, 1,156)
**Targeted** **(*P* = 0.2)**	269	62.2	875 (232, 1,521)	1,995 (913, 4,663)	541 (58, 1,248)
**Untargeted** **(*P* = 0)**	215	50.0	886 (310, 1,487)	1,602 (733, 3,745)	675 (102, 1,523)

^1^For the cost-effectiveness analysis, both DALYs and costs (in 2024 USD) are discounted at a rate of 3% per year.

^2^Incremental cost is assessed relative to the no-ACF scenario.

### Sensitivity analyses

When we assumed that ACF reduced transmission among individuals identified with TB by 75% (vs. 50% on the main analysis), ICERs fell by approximately 30% compared to the main analysis, ranging from $300 (95% CrI [dominant, $800]) with *P* = 1, to $500 (95% CrI [$20, $1,200]) with *P* = 0 per DALY averted. In a sensitivity analysis that assumed a higher baseline TB prevalence (452 per 100,000, versus 215 per 100,000 in the main analysis), ICERs fell by approximately 50% compared to the main analysis, ranging from $200 (95% CrI [dominant, $500]) (*P* = 1) to $300 (95% CrI [dominant, $800]) (*P* = 0) per DALY averted. In a sensitivity analysis that excluded transmission benefits, ICERs were 2.5–3.0 times the values estimated in the main analysis, ranged from $1,200 (95% CrI [$600, $2,100]) (*P* = 1) to $1,800 (95% CrI [$900, $3,200]) (*P* = 0) per DALY averted. Additional results for these sensitivity analyses are shown in Table M in S1 Appendix.

## Discussion

This study shows that geographically targeted ACF using ARTI estimates derived from a convenience sampling approach for *Mtb* immunoreactivity in young children can result in greater health gains and efficiency compared to untargeted ACF. In Blantyre, Malawi—a setting with a high burden of TB and HIV—we found that differences in community-level ARTI, as measured through *Mtb* immunoreactivity surveys, could help prioritize areas for diagnostic interventions more effectively than untargeted approaches. Combining novel approaches to TB surveillance (convenience *Mtb* immunoreactivity sampling among under 5-year-old children) with targeted ACF therefore offers an approach to increase the effectiveness and efficiency of TB screening in high-burden settings.

Prioritizing where to implement ACF has been a long-standing challenge for TB programs [3]. Although modeling analyses have suggested that targeted ACF could offer substantial efficiency gains in reducing transmission [33], systematic reviews of implementation studies have identified only a small number of studies that evaluated the effectiveness of targeted TB interventions [34,35], likely because of the difficulty in identifying a suitable epidemiological marker to guide targeting. Our findings suggest that ARTI data could fill this gap, offering a feasible and practical epidemiological marker for directing ACF implementation.

For programs planning to implement ACF, how to best deploy ACF services is a central question. In our analysis, compared to PCF alone, ACF improved life expectancy, reduced TB-attributable mortality, and averted 767 DALYs per 100,000 individuals screened. Efficiency gains were observed when comparing targeted to untargeted ACF. For example, providing ACF to approximately half of the population through targeted ACF could find 80% of all TB cases potentially identifiable through ACF, with more favorable ICERs compared to untargeted ACF. These findings highlight the potential value of geographic targeting in enhancing the efficiency of ACF implementation under real-world constraints on population coverage.

While targeted ACF improved efficiency relative to untargeted ACF, the absolute cost-effectiveness of the intervention remains a critical consideration [36,37]. For Malawi, published estimates suggest that cost-effectiveness threshold may lie between 33% and 44% of per-capita GDP (equivalent to $205 to $274 in 2025 USD) [38]. A recent government report proposed a more stringent threshold of $66 per DALY averted [39]. By these benchmarks, the ICERs estimated for both targeted and untargeted ACF—ranging from $400 to $700 per DALY averted—would not meet conventional definitions of cost-effectiveness, and represent a substantial cost relative to available healthcare funding Malawi, where per capita health expenditure was approximately US$41 in 2023. However, for infectious diseases like TB such thresholds may underestimate longer-term population benefits, including transmission reduction, mitigation of future risks of drug-resistant disease, and progress toward elimination goals. Moreover, the cost-effectiveness of ACF could improve under settings with higher TB prevalence or with reduced implementation costs.

These findings suggest that although ACF is a high-cost intervention, its efficiency can be substantially improved through geographic prioritization, potentially offering a higher return on investment in settings where ACF is already being implemented—as is the case in Blantyre and across Malawi. Although this study used ARTI estimates to guide targeted ACF, the modeling framework is data-source agnostic and can be parameterized using alternative proxies for TB burden, including case notification rates, genomic risk predictions, or community prevalence estimates. Each of these other data sources representing alternative possible approaches for targeting ACF.

A key contribution of this study lies in demonstrating the potential of using *Mtb* immunoreactivity survey data as a basis for targeting ACF. In many high-burden urban settings, including Blantyre, TB notification data may not accurately reflect underlying transmission risk due to variations in health system access, diagnostic capacity, or health-seeking behavior [4]. Historically, ARTI data have been widely used to track TB epidemiology and inform public health policy and planning, including prioritization of interventions such as case finding, preventive therapy, and vaccination strategies [40–44]. These surveys have been conducted less frequently in recent years, with greater priority being given to surveys of TB disease prevalence for tracking changes in TB epidemiology. However, compared to TB prevalence surveys immunoreactivity surveys are considerably less resource-intensive to implement, and can be feasibly integrated into existing surveillance systems, particularly within primary care settings [45]. For these reasons, there have been calls to expand the use of immunoreactivity surveys to supplement other methods for tracking TB burden. For this study, the cost of immunoreactivity survey implementation was excluded from the analysis, as the survey data were collected through a separate research study that was embedded within primary care visits, and not as part of the intervention being evaluated. While providing potentially valuable data, *Mtb* immunoreactivity surveys can introduce a number of challenges, including the feasibility and acceptability of testing young children, which were not examined in this analysis. Moreover, multiple factors could weaken the relationship between immunoreactivity survey estimates and true TB prevalence, including statistical uncertainty from small sample sizes, differential sampling bias across clusters, imperfect test performance, and uncertainty in the quantitative relationship between immunoreactivity among children and population TB prevalence. We tested the implications of these factors through varying the predictive power of ARTI, but more research on this relationship is needed.

Our model projected that ACF substantially reduced TB-attributable deaths, thereby increasing life expectancy among both people with and without HIV, underscoring its potential value in mitigating excess TB-associated mortality. This contrasts with findings from the TREATS study in Zambia and South Africa, which found no significant reduction in TB prevalence following a community-based intervention combining HIV testing, linkage to care, and TB symptom screening [46]. However, there are several differences between our study and the TREATS model, including differences in the approach for screening (TREATS primarily relied on symptom screening, whereas our ACF strategy also used chest X-ray with computer-aided detection for individuals without symptoms), and targeting (our ARTI-guided approach allowed fine-scale targeting of high-transmission neighborhoods, with potentially greater impact on transmission). Furthermore, ACF may mitigate long-term morbidity associated with post-TB sequelae, which are increasingly recognized as major contributors to TB disease burden [22,47]. These sequelae are often underdiagnosed and underreported yet contribute significantly to the burden of DALYs in high TB-burden settings [22]. By allowing earlier identification and initiation of treatment, ACF may prevent progression to severe TB disease and reduce the likelihood of permanent impairment. These delayed outcomes may not be captured by empirical data on short-term ACF outcomes.

At the cluster level, we observed substantial variation in the economic efficiency of ACF, with high-ARTI clusters generally achieving lower ICERs. These findings support a phased approach to ACF scale-up: initiating ACF in high-ARTI clusters and expanding outward as resources allow. However, interpretation of value for money should consider not only cost per DALY but also health system priorities, budget constraints, and equity considerations, particularly in resource-limited settings [3,37].

This study has several limitations. First, although our simulations assumed a correlation between ARTI and active TB disease prevalence, the strength of this relationship may vary depending on factors such as age, immune status, test performance, and sampling uncertainty. In addition, ARTI estimates themselves are subject to uncertainty, particularly in clusters with zero positives, which may reflect limited sample size rather than true absence of infection. To explore this, we modeled a range of predictive power scenarios; nonetheless, further empirical validation of the link between ARTI and TB prevalence is needed [44,48,49]. Second, we computed the number of TB cases that could be averted by ACF based on an estimate of the reproductive number for Blantyre and an assumption that early diagnosis via ACF would avert approximately half of the transmission associated with each TB case. However, there is substantial uncertainty about the effects of ACF on transmission, and sensitivity analyses that tested optimistic and pessimistic transmission assumptions found that cost-effectiveness ratios were sensitive to the magnitude of transmission effects. Third, we examined a scenario in which immunoreactivity survey data were already available from an earlier study. The cost of collecting immunoreactivity survey data is a factor that could be influential for cost-effectiveness, and so while excluded from this analysis these costs would need to be considered in other settings where these data are not available. For this reason, research is needed on how to best conduct these surveys, how to make them more efficient, and the frequency with which they need to be repeated. Fourth, we only examined one ACF diagnostic algorithm, yet different approaches have been proposed [50], and new technologies may soon be available that lower the cost of testing [51]. Fifth, in this study we examined the impact and cost-effectiveness of a single round of ACF. However, in some settings ACF will be implemented as repeated rounds, with each round generating additional health benefits by identifying further people with TB. For ACF implemented as multiple rounds, the long-term health effects will depend on changes in disease prevalence over time and resulting dynamic effects on TB transmission [52]. We did not explicitly model repeated ACF rounds, and the incremental costs and benefits of such strategies may differ from those presented here. Lastly, this study was conducted in a setting of co-prevalent HIV and TB, and the health impact and cost-effectiveness of ACF will vary according to the prevalence of these conditions.

Evidence describing even modest differences in transmission can inform more strategic and efficient ACF delivery. Building on data sources like convenience-based immunoreactivity surveys, this approach can be embedded in national surveillance systems and adapted to other high-burden settings. While targeted ACF improves efficiency, it raises equity concerns and cost implications—particularly around prioritization and long-term ART provision following extended survival. These downstream costs, though linked to positive outcomes, should be considered in programmatic planning.

In conclusion, geographically targeted TB ACF directed by ARTI data from convenience surveys of *Mtb* immunoreactivity offers a data-driven strategy to improve the efficacy and effectiveness of community-based TB screening. Even in settings with relatively uniform burden, immunoreactivity data can support more targeted resource allocation and strengthen the case for scaling up ACF. Embedding targeted approaches within existing systems—while considering equity and long-term costs—may improve the effectiveness and sustainability of TB programs.

## Supporting information

S1 Appendix Supplementary figures and tables. (PDF)

S2 Appendix Inclusivity in global research checklist. (PDF)

## Abbreviations

ACF: Active case finding
AI: Artificial Intelligence
ARTI: Annual Risk of TB Infection
CrI: Credible Interval
DALY: Disability-Adjusted Life Year
ICERs: Incremental Cost-Effectiveness Ratios
IGRAs: Interferon-Gamma Release Assays
LE: Life Expectancies
NTLEP: National Tuberculosis and Leprosy Elimination Program
PCF: Passive Case Finding
YLDs: Years Lived with Disability
YLLs: Years of Life Lost
