# Supplementary material for: Potential health impacts and costs of active case finding guided by Mycobacterium tuberculosis immunoreactivity survey results in Blantyre, Malawi: A mathematical modeling study
Source: PLoS Med. 2026 Oct 7;23(10):e1005272. doi: 10.1371/journal.pmed.1005272 (PMC13657404; doi:10.1371/journal.pmed.1005272)
Supplement: Supplementary file 1 [file pmed.1005272.s001.pdf]

[APPENDIX] Potential health impacts and costs of active case finding guided by *Mycobacterium tuberculosis* immunoreactivity survey results in Blantyre, Malawi

LIST OF LEGENDS

[Figures]

Fig A: Schematic of the model structure used for simulating individual health outcomes.

Fig B: Cumulative costs and health benefits of expanding ACF under varying ARTI predictive power assumptions.

[Tables]

Table A: Key model parameters.

Table B: Cost parameter inputs.

Table C: Parameters for the ACF diagnostic algorithm.

Table D: Baseline characteristics in each cluster.

Table E: Estimated years of healthy life lost due to disability (YLDs).

Table F: Estimated years of life lost due to premature mortality (YLLs).

Table G: Estimated TB-attributable deaths (as a percentage of total deaths, %).

Table H: Cost-effectiveness of targeted ACF by cluster (ARTI P^*=0.8).

Table I: Cost-effectiveness of targeted ACF by cluster (ARTI P^*=0.6).

Table J: Cost-effectiveness of targeted ACF by cluster (ARTI P^*=0.4).

Table K: Cost-effectiveness of targeted ACF by cluster (ARTI P^*=0.2).

Table L: Cost-effectiveness of untargeted ACF by cluster (ARTI P^*=0).

Table M: Sensitivity analyses: cost-effectiveness of targeted ACF vs. untargeted ACF at 50% cluster coverage.

[APPENDIX] Potential health impacts and costs of active case finding guided by Mycobacterium tuberculosis immunoreactivity survey results in Blantyre, Malawi

Fig A: Schematic of the model structure used for simulating individual health outcomes (reproduced from Can et al., 2025 [1]).

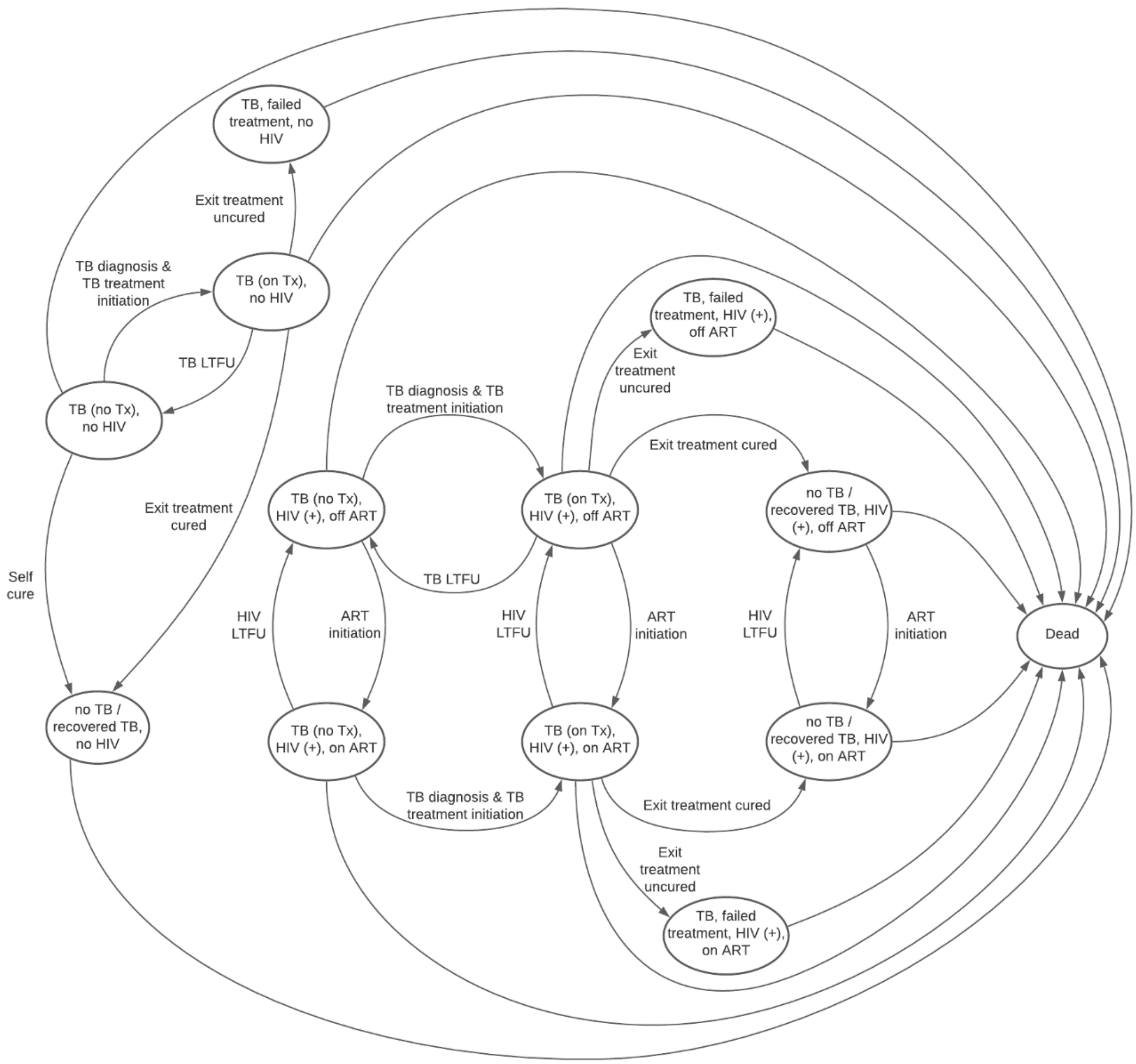

Fig B: Cumulative costs and health benefits of expanding ACF under varying ARTI predictive power assumptions.

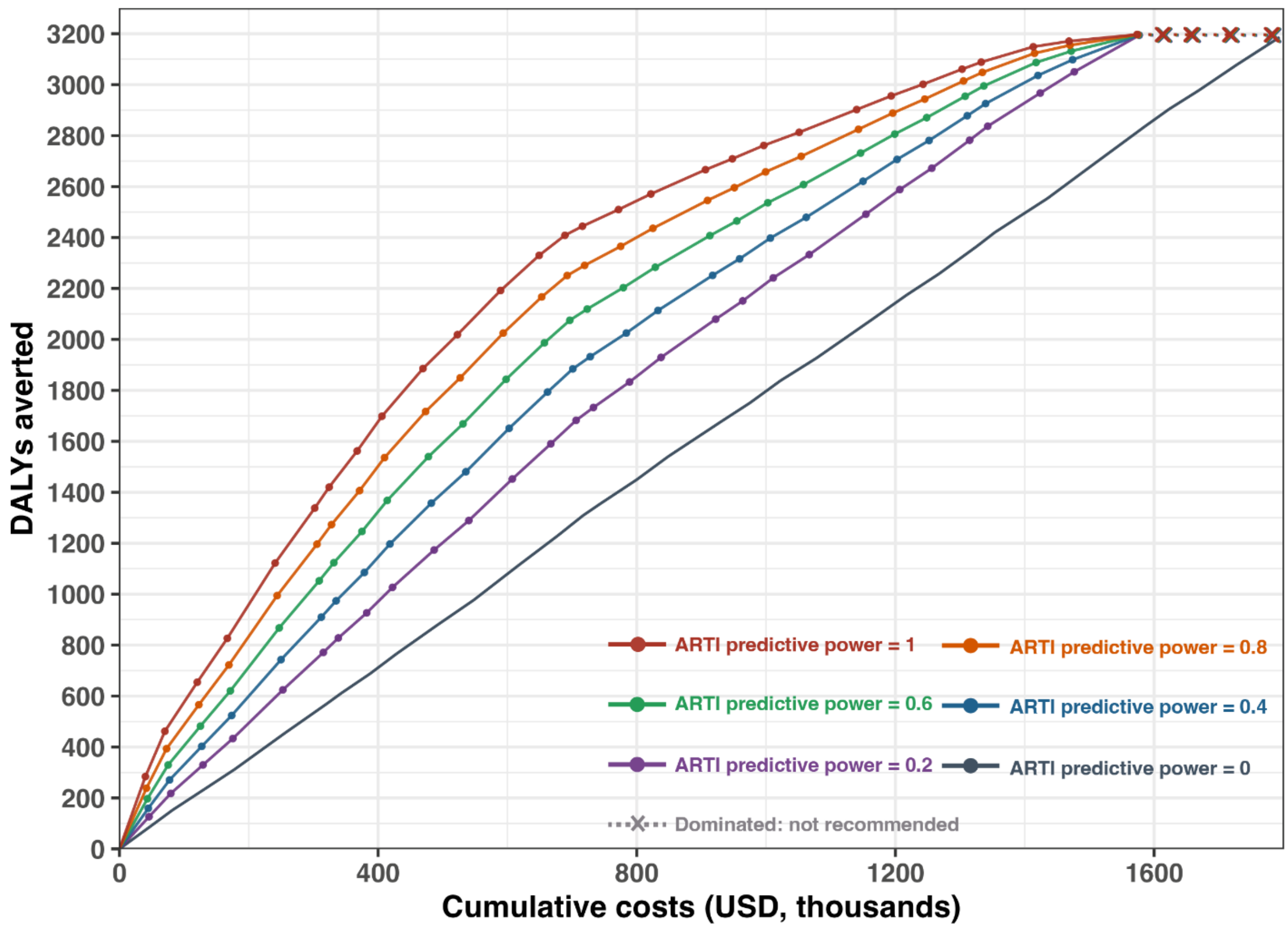

*Dominated points (marked ×) represent clusters where ACF was more costly and less effective than the *PCF-Only* strategy.

[APPENDIX] Potential health impacts and costs of active case finding guided by Mycobacterium tuberculosis immunoreactivity survey results in Blantyre, Malawi

Table A: Key model parameters.

Parameter	Value/Assumption (95% interval)	Prior distribution	Source
Mortality			
Background (non-TB/non-HIV) annual mortality rate	Age- and sex-specific	Fixed values	[2,3]
Rate ratio for TB mortality by year of age (*reference age = 40*)	Age-specific	Fixed values	[2]
Annual rate of TB-specific mortality, for age = 40	0.29 (0.21, 0.40)	Gamma (37.84, 130.05)	[4]
Annual mortality rate due to treatment side effects (applied to TB treatment)	0.13 (0.80, 2.10) x 1e-3	Gamma (15.2, 11686)	[5]
Rate ratio for mortality due to lung damage	Fitted log-linear function ($\exp(\beta \times (1 - FEV1p))$), with parameter β = 1.75 (1.59, 1.91)	Normal (1.753, 0.082)	[6]
HIV-specific annual mortality rate, off ART	Log-linear function of CD4 cell count ($\exp(\beta_0 + \beta_1 \times CD4)$), with parameters β_0 = -0.011 (-0.012, -0.009) β_1 = -0.175 (-0.367, 0.017)	β_0: Normal (-0.011, 0.0006) β_1: Normal (-0.175, 0.098)	[7]
HIV-specific annual mortality rate, on ART	Log-linear function of CD4 cell count ($\exp(\beta_0 + \beta_1 \times CD4)$), with parameters β_0= -0.0058 (-0.013, 0.0012) β_1= -3.533 (-5.538, -1.53)	β_0: Normal (-0.0058, 0.0036) β_1: Normal (-3.533, 1.02)	[8]
TB treatment and cure			
Probability of cure with TB treatment completion	0.94 (0.91, 0.96)	Beta (322.3, 20.6)	[9]
Annual rate of returning to TB care following discontinuation or failure, off ART	2.0 (1.0, 4.0)	Gamma (6.7, 3.3)	[1]
Annual rate of returning to TB care following discontinuation or failure, on ART	4.0 (2.0, 8.0)	Gamma (6.656, 1.664)	[1]
Probability of LTFU prior to TB treatment initiation	0.14 (0.07, 0.21)	Beta (12.91, 79.30)	[1]
Annual rate of stopping TB care (LTFU)	0.13 (0.10, 0.20)	Gamma (27.1, 208.8)	[10]
Probability of cure at LTFU	0.2 (0.05, 0.35)	Beta (5.1, 20.4)	[1]
TB regimen duration (weeks)	26	Fixed	[1]
Annual base rate of self-cure, for untreated active TB	0.37 (0.29, 0.47)	Gamma (63.688, 172.596)	[1]
TB lung damage			
Mean FEV1 among individuals with TB	63.1% (53.1, 73.1)	Beta (55.1, 32.5)	[11–16]
Standard deviation of FEV1 among individuals with TB	15%	Fixed	[11–13,15,16]
Rate of FEV1 decline with untreated TB (percentage points per week)	*HIV-negative:* 0.70 (0.55, 0.85)	Gamma (53.8, 35.9)	[11–17]

[APPENDIX] Potential health impacts and costs of active case finding guided by Mycobacterium tuberculosis immunoreactivity survey results in Blantyre, Malawi

Parameter	Value/Assumption (95% interval)	Prior distribution	Source
	HIV-positive: 1.52 (1.20, 2.00)	Gamma (83.5, 119.3)	
Rate of FEV1 increase following TB cure, on treatment (percentage points per week)	0.44 (0.34, 0.54)	Gamma (61.3, 139.3)	[11–13,16,18–21]
Rate of FEV1 increase following TB cure, not on treatment (percentage points per week)	0.096 (0.066, 0.126)	Gamma (39.2, 407.9)	[11,20,22]
Maximum possible recovery of FEV1 following TB cure (fraction of 100% minus minimum FEV1 value)	0.42 (0.30, 0.60)	Beta (16.8, 23.3)	[11–13,15,16,20,22]
HIV natural history and treatment			
Rate of decline in CD4 count without ART (cells/mm^3 per week)	1.17 (0.89, 1.56)	Gamma (46.7, 39.9)	[23]
Rate of increase in CD4 count with ART (cells/mm^3 per week)	Log-linear function of time since ART initiation ($\delta_1(1 - \exp(-\exp(\delta_2 \times t_{ART})))$, with parameters δ_1 = 247 (245, 250) δ_1 = -6.96 (-7.02, -6.90)	δ_1: Normal (247.2, 1.32) δ_2: Normal (-6.96, 0.029)	[24]
Annual rate of loss-to-follow-up from ART	0.064 (0.044, 0.094)	Gamma (25.0, 390.6)	[25]
Time since ART initiation for individuals receiving ART at presentation	Sampled from a uniform distribution between 3 months and 5 years	Uniform (0.25, 5.0)	[1]
Disability weights and discounting			
Post-TB disability weights, by FEV1 category	*FEV1 ≥ 80%:* 0.005 (0.003, 0.010) *80% > FEV1 ≥ 50%:* 0.047 (0.024, 0.071) *50% > FEV1:* 0.366 (0.275, 0.458)	Beta (7.6, 1517) Beta (14.4, 292.5) Beta (38.4, 66.6)	[26,27]
TB disability weights, by FEV1 category	*FEV1 ≥ 80%:* 0.092 (0.046, 0.138) *80%> FEV1 ≥ 50%:* 0.135 (0.101, 0.203) *80% > FEV1:* 0.453 (0.340, 0.566)	Beta (13.7, 135.1) Beta (23.0, 147.3) Beta (33.1, 40.0)	[26–28]
HIV disability weights, by CD4 category (CD4 count values are in cells/μL)	*CD4 ≥ 350, no ART:* 0.012 (0.006, 0.023) *350 > CD4 ≥ 200, no ART:* 0.274 (0.184, 0.377) *200 > CD4, no ART:* 0.582 (0.406, 0.743) *On ART:* 0.078 (0.052, 0.111)	Beta (7.4, 607.6) Beta (22.0, 58.4) Beta (18.3, 13.1) Beta (24.5, 289.8)	[28]
Disability weight of TB treatment due to a false-positive TB diagnosis	0.049 (0.031, 0.072)	Beta (20.7, 400.8)	[28]
Discount rate	0.03	Fixed value	[1,29]

Table B: Cost parameter inputs.

Parameter	Value/Assumption (95% interval)	Prior distribution	Source
Health services costs			
Costs for TB diagnosis (per person, USD)	19.09 (13.38, 24.80)	Gamma (42.8, 2.24)	[30]
Costs for TB treatment (per month, USD)	43.80 (24.50, 75.80)	Gamma (11.0, 0.25)	[31]
Drug costs for TB treatment (per month, USD)	6.00 (3.00, 9.00)	Gamma (15.2, 2.53)	Malawi NTLEP report 2024 (unpublished)
Antiretroviral therapy (ART) costs (per month, USD)	21.67 (14.75, 28.67)	Gamma (37.1, 1.71)	[32]
Active case finding (per person, USD)	7.00 (4.00, 12.00)	Gamma (11.6, 1.66)	NTLEP, personal communication, 2025
Patient costs			
Patient productivity losses related to post-TB sequelae (per month, USD)	0.60 (0.30, 1.36)	Gamma (4.76, 43.7)	[20]
Patient direct non-medical costs of untreated TB (per month, USD)	8.34 (5.75, 12.11)	Gamma (26.2, 3.14)	[33,34]
Patient indirect costs of untreated TB (per month, USD)	9.42 (6.81, 12.57)	Gamma (41.0, 4.35)	[33,34]
Patient direct non-medical costs, TB treatment (per month, USD)	8.34 (5.75, 12.11)	Gamma (26.2, 3.14)	[33,34]
Patient indirect costs, TB treatment (per month, USD)	9.42 (6.81, 12.57)	Gamma (41.0, 4.35)	[33,34]

Table C: Parameters for the ACF diagnostic algorithm.

Parameter	Value/Assumption (95% interval)	Prior distribution	Source
Sensitivity, symptom screen (prolonged cough)	42.0% (31.5, 52.5)	Beta (35.0, 48.4)	[35]
Sensitivity, chest X-ray (any abnormality)	94.0% (91.0, 97.0)	Beta (223, 14.2)	[35]
Sensitivity, Xpert MTB/RIF	84.7% (78.6, 89.9)	Beta (130, 23.5)	[36,37]
Specificity, symptom screen (any cough)	94.0% (92.5, 95.5)	Beta (902, 57.5)	[35]
Specificity, chest X-ray (any abnormality)	89.0% (86.3, 91.8)	Beta (440, 54.4)	[35]
Specificity, Xpert MTB/RIF	99.80% (99.75, 99.85)	Beta (30521, 61.2)	[36,37]
Pre-treatment loss to follow-up	5.0% (2.5,7.5)	Beta (14.4, 273)	NTLEP, personal communication, 2025

Table D: Baseline characteristics in each cluster.

Cluster No.	Population	HIV prevalence among TB pop. (%)	*Mtb* immune-reactivity prevalence (%)	ARTI* (%)	TB disease prevalence (per 100,000), under different assumptions for the predictive power of ARTI (*P*)					
					P=1	P=0.8	P=0.6	P=0.4	P=0.2	P=0
1	9537	44	7.7	3.1	254	262	265	262	255	215
2	7675	73	3.9	1.5	125	149	173	198	221	215
3	5897	68	3.6	1.7	134	157	180	203	224	215
4	7524	48	4.1	1.8	145	167	189	209	228	215
5	7198	50	20	8.6	701	588	486	394	312	215
6	5643	53	0	0	0	0	0	0	0	215
7	7837	54	4.4	1.5	123	146	171	196	220	215
8	6573	66	12	5.9	480	435	387	338	290	215
9	8946	50	3.5	1.5	118	141	167	193	219	215
10	11433	69	3	1.2	98	122	149	179	210	215
11	12070	65	4.7	1.8	145	167	189	209	228	215
12	6417	45	12.1	4.6	374	356	333	306	275	215
13	7982	65	4.7	1.9	150	172	193	213	230	215
14	15231	48	1	0.4	31	48	75	113	167	215
15	8054	47	1.5	0.6	49	70	98	136	183	215
16	6476	36	6.2	2.6	211	225	236	243	245	215
17	7904	59	8.5	3.8	305	302	295	282	264	215
18	11002	40	7	3.4	275	278	277	271	259	215
19	7027	67	15.2	6.2	503	452	398	345	292	215
20	3606	50	14.3	5	405	379	350	316	280	215
21	6390	61	12.8	5	404	379	349	316	280	215
22	9435	56	13.4	5.1	411	384	353	318	281	215
23	7233	50	4.3	1.6	129	152	176	200	223	215
24	11967	51	12.5	5.4	440	405	367	327	284	215
25	5369	48	17.1	7.2	586	510	436	366	301	215
26	12976	57	3.7	1.5	124	148	172	197	221	215
27	9715	55	10.8	4.3	345	334	318	297	271	215
28	7038	67	3.6	1.5	120	144	169	195	219	215
29	4463	41	3.5	1.3	108	132	158	186	215	215
30	3934	60	5.3	2	164	184	203	220	234	215
31	6323	73	0	0	0	0	0	0	0	215
32	8574	62	0	0	0	0	0	0	0	215
33	9263	69	0	0	0	0	0	0	0	215

*ARTI refers to the annual risk of tuberculosis infection.

Table E: Estimated years of healthy life lost due to disability (YLDs).

Category		YLDs under ACF scenario	YLDs under PCF scenario	Incremental Difference
Individuals with TB	HIV–negative	1.0 (0.5, 1.9)	1.7 (0.8, 3.4)	-0.6 (-1.6, -0.2)
Individuals with TB	HIV–positive	2.1 (1.1, 2.9)	2.2 (1.1, 3.1)	-0.0 (-0.4, 0.2)
Individuals without TB	HIV–negative	$5.9e^{-6}$ ($3.2e^{-6}$, $9.5e^{-6}$)	0.0 (0.0, 0.0)	$5.9e^{-6}$ ($3.2e^{-6}$, $9.5e^{-6}$)
Individuals without TB	HIV–positive	2.1 (1.0, 2.8)	2.1 (1.0, 2.8)	$4.1e^{-6}$ ($-3.0e^{-7}$, $8.4e^{-6}$)

Table F: Estimated years of life lost due to premature mortality (YLLs).

Category		YLLs under ACF scenario	YLLs under PCF scenario	Incremental Difference
Individuals with TB	HIV–negative	9.2 (6.2, 12.5)	14.4 (9.3, 20.3)	-5.2 (-8.3, -2.7)
Individuals with TB	HIV–positive	33.9 (28.9, 41.9)	36.7 (31.8, 43.3)	-2.8 (-4.9, -1.0)
Individuals without TB	HIV–negative	$4.6e^{-5}$ ($7.1e^{-6}$, $1.0e^{-4}$)	0.0 (0.0, 0.0)	$4.6e^{-5}$ ($7.1e^{-6}$, $1.0e^{-4}$)
Individuals without TB	HIV–positive	29.6 (23.2, 40.1)	29.6 (23.2, 40.1)	$1.7e^{-5}$ ($8.1e^{-7}$, $5.1e^{-5}$)

Table G: Estimated TB-attributable deaths (as a percentage of total deaths, %).

Category		Deaths under ACF scenario	Deaths under PCF scenario	Incremental Difference
Individuals with TB	HIV–negative	8.9 (5.1, 14.7)	16.2 (8.5, 27.7)	-7.2 (-13.4, -3.3)
Individuals with TB	HIV–positive	22.3 (14.3, 33.1)	36.5 (22.6, 55.6)	-14.2 (-23.6, -7.3)

Table H: Cost-effectiveness of targeted ACF by cluster (ARTI P^{*}=0.8).

Cluster No.	Population	ARTI	Incremental cost (2024 USD)	DALYs averted	ICER (USD per DALY averted)
5	7198	0.0862	41259	239	173
25	5369	0.0721	30726	155	198
19	7027	0.0619	49561	173	287
8	6573	0.0591	46025	156	295
24	11967	0.0541	73917	273	271
22	9435	0.0506	61016	202	302
20	3606	0.0498	22203	77	288
21	6390	0.0497	43078	133	323
12	6417	0.046	38236	130	294
27	9715	0.0425	62960	181	348
17	7904	0.0375	52919	132	400
18	11002	0.0338	65934	176	375
1	9537	0.0313	58940	142	415
16	6476	0.0259	39129	84	465
30	3934	0.0202	26788	40	670
13	7982	0.0185	55372	75	740
4	7524	0.0178	49325	71	698
11	12070	0.0178	83788	110	764
3	5897	0.0165	41289	50	823
23	7233	0.0159	47885	62	774
2	7675	0.0154	54548	61	893
26	12976	0.0153	87919	106	828
7	7837	0.0151	52662	64	825
28	7038	0.0148	49047	55	892
9	8946	0.0145	59422	71	836
29	4463	0.0133	28979	34	860
10	11433	0.012	80008	75	1064
15	8054	0.006	54444	32	1719
14	15231	0.0038	103837	41	2510
6	5643	0	39110	-0.15	Dominated
31	6323	0	43817	-0.17	Dominated
32	8574	0	59422	-0.23	Dominated
33	9263	0	64193	-0.25	Dominated
Total	266710	-	1767760	3198	677

$^{*}P$ denotes predictive power

[APPENDIX] Potential health impacts and costs of active case finding guided by Mycobacterium tuberculosis immunoreactivity survey results in Blantyre, Malawi

Table I: Cost-effectiveness of targeted ACF by cluster (ARTI P^*=0.6).

Cluster No.	Population	ARTI	Incremental cost (2024 USD)	DALYs averted	ICER (USD per DALY averted)
5	7198	0.0862	42764	197	217
25	5369	0.0721	31662	133	239
19	7027	0.0619	49460	153	324
8	6573	0.0591	45973	139	331
24	11967	0.0541	74763	247	303
22	9435	0.0506	61373	185	331
20	3606	0.0498	22422	71	316
21	6390	0.0497	43173	123	351
12	6417	0.046	38635	122	318
27	9715	0.0425	63174	172	367
17	7904	0.0375	52966	129	411
18	11002	0.0338	65980	175	377
1	9537	0.0313	58861	144	410
16	6476	0.0259	38841	88	439
30	3934	0.0202	26739	44	606
13	7982	0.0185	55379	84	658
4	7524	0.0178	48954	80	612
11	12070	0.0178	83806	124	675
3	5897	0.0165	41352	58	717
23	7233	0.0159	47532	72	663
2	7675	0.0154	54773	71	769
26	12976	0.0153	87585	124	707
7	7837	0.0151	52381	75	702
28	7038	0.0148	49095	65	760
9	8946	0.0145	58961	84	703
29	4463	0.0133	28588	40	707
10	11433	0.012	80182	92	870
15	8054	0.006	53885	45	1207
14	15231	0.0038	102909	64	1608
6	5643	0	39110	-0.15	Dominated
31	6323	0	43817	-0.17	Dominated
32	8574	0	59422	-0.23	Dominated
33	9263	0	64193	-0.25	Dominated
Total	266710	-	1768708	3198	678

$^*P$ denotes predictive power

Table J: Cost-effectiveness of targeted ACF by cluster (ARTI P^*=0.4).

Cluster No.	Population	ARTI	Incremental cost (2024 USD)	DALYs averted	ICER (USD per DALY averted)
5	7198	0.0862	44117	160	276
25	5369	0.0721	32552	111	292
19	7027	0.0619	49358	132	374
8	6573	0.0591	45920	121	378
24	11967	0.0541	75669	220	345
22	9435	0.0506	61770	167	370
20	3606	0.0498	22669	64	353
21	6390	0.0497	43279	111	389
12	6417	0.046	39111	112	350
27	9715	0.0425	63452	161	395
17	7904	0.0375	53044	123	430
18	11002	0.0338	66218	171	388
1	9537	0.0313	58920	143	413
16	6476	0.0259	38659	91	424
30	3934	0.0202	26696	48	558
13	7982	0.0185	55385	93	597
4	7524	0.0178	48602	89	547
11	12070	0.0178	83824	138	608
3	5897	0.0165	41414	65	637
23	7233	0.0159	47179	81	579
2	7675	0.0154	54999	81	675
26	12976	0.0153	87246	142	615
7	7837	0.0151	52096	86	608
28	7038	0.0148	49144	74	660
9	8946	0.0145	58484	97	602
29	4463	0.0133	28173	48	591
10	11433	0.012	80374	111	726
15	8054	0.006	53150	62	861
14	15231	0.0038	101555	97	1047
6	5643	0	39110	-0.15	Dominated
31	6323	0	43817	-0.17	Dominated
32	8574	0	59422	-0.23	Dominated
33	9263	0	64193	-0.25	Dominated
Total	266710	-	1769598	3197	678

$^*P$ denotes predictive power

Table K: Cost-effectiveness of targeted ACF by cluster (ARTI P^*=0.2).

**Cluster No.**	**Population**	**ARTI**	**Incremental cost (2024 USD)**	**DALYs averted**	**ICER (USD per DALY averted)**
5	7198	0.0862	45309	127	358
25	5369	0.0721	33379	91	365
19	7027	0.0619	49258	112	440
8	6573	0.0591	45866	104	442
24	11967	0.0541	76609	191	401
22	9435	0.0506	62196	147	422
20	3606	0.0498	22935	57	404
21	6390	0.0497	43394	98	441
12	6417	0.046	39650	100	395
27	9715	0.0425	63785	147	435
17	7904	0.0375	53153	116	460
18	11002	0.0338	66653	163	408
1	9537	0.0313	59125	138	427
16	6476	0.0259	38603	92	420
30	3934	0.0202	26662	51	526
13	7982	0.0185	55390	100	554
4	7524	0.0178	48292	97	499
11	12070	0.0178	83840	150	559
3	5897	0.0165	41471	72	577
23	7233	0.0159	46848	91	517
2	7675	0.0154	55215	91	606
26	12976	0.0153	86922	159	546
7	7837	0.0151	51822	96	538
28	7038	0.0148	49191	84	585
9	8946	0.0145	58016	110	527
29	4463	0.0133	27751	55	505
10	11433	0.012	80576	130	618
15	8054	0.006	52212	83	625
14	15231	0.0038	99632	144	693
6	5643	0	39110	-0.15	Dominated
31	6323	0	43817	-0.17	Dominated
32	8574	0	59422	-0.23	Dominated
33	9263	0	64193	-0.25	Dominated
Total	266710	-	1770297	3196	679

$^*P$ denotes predictive power

Table L: Cost-effectiveness of untargeted ACF by cluster (ARTI P^*=0).

Cluster No.	Population	ARTI	Incremental cost (2024 USD)	DALYs averted	ICER (USD per DALY averted)
5	7198	0.0862	46732	87	536
25	5369	0.0721	34475	65	528
19	7027	0.0619	49111	82	597
8	6573	0.0591	45785	77	594
24	11967	0.0541	78153	144	541
22	9435	0.0506	62942	113	558
20	3606	0.0498	23411	44	537
21	6390	0.0497	43600	76	576
12	6417	0.046	40705	78	519
27	9715	0.0425	64516	116	554
17	7904	0.0375	53457	94	569
18	11002	0.0338	68279	136	504
1	9537	0.0313	60217	117	516
16	6476	0.0259	39383	80	489
30	3934	0.0202	26709	47	572
13	7982	0.0185	55386	94	591
4	7524	0.0178	48506	91	531
11	12070	0.0178	83829	142	592
3	5897	0.0165	41446	69	602
23	7233	0.0159	46961	88	537
2	7675	0.0154	55159	89	622
26	12976	0.0153	87002	155	562
7	7837	0.0151	51882	94	552
28	7038	0.0148	49183	82	597
9	8946	0.0145	58082	108	537
29	4463	0.0133	27747	55	504
10	11433	0.012	80605	133	605
15	8054	0.006	51585	98	526
14	15231	0.0038	97939	185	529
6	5643	0	37106	68	547
31	6323	0	45326	73	620
32	8574	0	58613	101	578
33	9263	0	65439	108	607
Total	266710	-	1779268	3189	683

$^*P$ denotes predictive power

Table M: Sensitivity analyses: cost-effectiveness of targeted and untargeted ACF vs. PCF-only at 50% cluster coverage.

	**i) 75% transmission reduction**			**ii) Higher TB prevalence estimates**		
	**Incremental cost[2] in thousand**	**DALYs averted**	**ICER compared to no ACF**	**Incremental cost[2] in thousand**	**DALYs averted**	**ICER compared to no ACF**
**Targeted (*P*=1)**	681 (-358, 1,440)	3,391 (1,478, 8,065)	271 (dominant, 780)	780 (-626, 1,866)	5,497 (2,518, 12,836)	191 (dominant, 537)
**Targeted (*P*=0.8)**	693 (-313, 1,425)	3,224 (1,405, 7,667)	288 (dominant, 812)	786 (-562, 1,829)	5,227 (2,394, 12,204)	201 (dominant, 558)
**Targeted (*P*=0.6)**	706 (-252, 1,419)	3,034 (1,322, 7,214)	310 (dominant, 859)	794 (-476, 1,785)	4,919 (2,253, 11,485)	214 (dominant, 584)
**Targeted (*P*=0.4)**	722 (-179, 1,419)	2,822 (1,229, 6,709)	337 (dominant, 914)	803 (-386, 1,742)	4,575 (2,096, 10,682)	231 (dominant, 609)
**Targeted (*P*=0.2)**	739 (-99, 1,416)	2,590 (1,127, 6,157)	373 (dominant, 984)	813 (-302, 1,713)	4,199 (1,923, 9,805)	252 (dominant, 651)
**Untargeted (*P*=0)**	776 (64, 1,409)	2,080 (905, 4,945)	478 (15, 1,199)	836 (-76, 1,626)	3,372 (1,545, 7,876)	316 (dominant, 778)

	**iii) Transmission benefits excluded (pessimistic assumption)**			**Main analysis (for comparison)**		
	**Incremental cost[2] in thousand**	**DALYs averted**	**ICER compared to no ACF**	**Incremental cost[2] in thousand**	**DALYs averted**	**ICER compared to no ACF**
**Targeted (*P*=1)**	1,215 (722, 1,844)	1,061 (650, 1,606)	1,199 (618, 2,118)	859 (91, 1,555)	2,613 (1,196, 6,104)	411 (20, 979)
**Targeted (*P*=0.8)**	1,200 (713, 1,824)	1,009 (618, 1,527)	1,247 (641, 2,208)	862 (124, 1,546)	2,484 (1,137, 5,804)	433 (26, 1,029)
**Targeted (*P*=0.6)**	1,184 (705, 1,791)	950 (582, 1,437)	1,307 (671, 2,327)	866 (158, 1,538)	2,338 (1,070, 5,462)	460 (34, 1,085)
**Targeted (*P*=0.4)**	1,166 (698, 1,762)	883 (541, 1,337)	1,385 (708, 2,463)	870 (197, 1,529)	2,174 (995, 5,080)	495 (44, 1,156)
**Targeted (*P*=0.2)**	1,146 (688, 1,741)	810 (496, 1,227)	1,485 (757, 2,635)	875 (232, 1,521)	1,995 (913, 4,663)	541 (58, 1,248)
**Untargeted (*P*=0)**	1,104 (662, 1,691)	650 (398, 985)	1,785 (899, 3,197)	886 (310, 1,487)	1,602 (733, 3,745)	675 (102, 1,523)

[1] For the cost-effectiveness analysis, both DALYs and costs (in 2024 USD) are discounted at a rate of 3% per year.
[2] Incremental cost is assessed relative to the no-ACF scenario.

References

1. Can MH, Sweeney S, Allwood BW, Dorman SE, Cohen T, Menzies NA. Implications of progressive lung damage and post-tuberculosis sequelae for the health benefits of prompt tuberculosis treatment in high HIV prevalence settings: a mathematical modelling analysis. The Lancet Global Health. 2025;13: e1240–e1249. doi:10.1016/S2214-109X(25)00114-7

2. Global Burden of Disease Collaborative Network. Global Burden of Disease Study 2019 (GBD 2019) Disease and Injury Burden 1990-2019. Institute for Health Metrics and Evaluation (IHME); 2020. doi:10.6069/P5WM-5A36

3. United Nations. World Population Prospects 2019 | Population Division. 2019 [cited 28 Feb 2026]. Available: https://www.un.org/development/desa/pd/news/world-population-prospects-2019-0

4. Tiemersma EW, Werf MJ van der, Borgdorff MW, Williams BG, Nagelkerke NJD. Natural History of Tuberculosis: Duration and Fatality of Untreated Pulmonary Tuberculosis in HIV Negative Patients: A Systematic Review. PLOS ONE. 2011;6: e17601. doi:10.1371/journal.pone.0017601

5. Saukkonen JJ, Cohn DL, Jasmer RM, Schenker S, Jereb JA, Nolan CM, et al. An official ATS statement: hepatotoxicity of antituberculosis therapy. Am J Respir Crit Care Med. 2006;174: 935–952. doi:10.1164/rccm.200510-1666ST

6. Duong M, Islam S, Rangarajan S, Leong D, Kurmi O, Teo K, et al. Mortality and cardiovascular and respiratory morbidity in individuals with impaired FEV1 (PURE): an international, community-based cohort study. Lancet Glob Health. 2019;7: e613–e623. doi:10.1016/S2214-109X(19)30070-1

7. Anglaret X, Minga A, Gabillard D, Ouassa T, Messou E, Morris B, et al. AIDS and Non-AIDS Morbidity and Mortality Across the Spectrum of CD4 Cell Counts in HIV-Infected Adults Before Starting Antiretroviral Therapy in Côte d'Ivoire. Clin Infect Dis. 2012;54: 714–723. doi:10.1093/cid/cir898

8. Kroeze S, Ondoa P, Kityo CM, Siwale M, Akanmu S, Wellington M, et al. Suboptimal immune recovery during antiretroviral therapy with sustained HIV suppression in sub-Saharan Africa. AIDS. 2018;32: 1043–1051. doi:10.1097/QAD.0000000000001801

9. Cox HS, Morrow M, Deutschmann PW. Long term efficacy of DOTS regimens for tuberculosis: systematic review. BMJ. 2008;336: 484–487. doi:10.1136/bmj.39463.640787.BE

10. World Health Organization. Global tuberculosis report 2020. 2020. Available: https://www.who.int/publications/i/item/9789240013131

11. Allwood BW, Maasdorp E, Kim GJ, Cooper CB, Goldin J, van Zyl-Smit RN, et al. Transition from Restrictive to Obstructive Lung Function Impairment During Treatment and Follow-Up of Active Tuberculosis. Int J Chron Obstruct Pulmon Dis. 2020;15: 1039–1047. doi:10.2147/COPD.S219731

12. Chesov D, Butov D, Reimann M, Heyckendorf J, Myasoedov V, Butov T, et al. Impact of lung function on treatment outcome in patients with TB. Int J Tuberc Lung Dis. 2021;25: 277–284. doi:10.5588/ijtld.20.0949

13. Radovic M, Ristic L, Ciric Z, Dinic-Radovic V, Stankovic I, Pejcic T, et al. Changes in respiratory function impairment following the treatment of severe pulmonary tuberculosis – limitations for the underlying COPD detection. Int J Chron Obstruct Pulmon Dis. 2016;11: 1307–1316. doi:10.2147/COPD.S106875

14. Maguire GP, Anstey NM, Ardian M, Waramori G, Tjitra E, Kenangalem E, et al. Pulmonary tuberculosis, impaired lung function, disability and quality of life in a high-burden setting. Int J Tuberc Lung Dis. 2009;13: 1500–1506.

15. Guessogo WR, Mandengue SH, Assomo Ndemba PB, Medjo UO, Minye EE, Ahmaidi S, et al. Physical and functional follow-up of tuberculosis patients in initial intensive phase of treatment in Cameroon using the 6-min walk test. J Exerc Rehabil. 2016;12: 333–339. doi:10.12965/jer.1632620.310

16. Ralph AP, Kenangalem E, Waramori G, Pontororing GJ, Sandjaja null, Tjitra E, et al. High morbidity during treatment and residual pulmonary disability in pulmonary tuberculosis: under-recognised phenomena. PLoS One. 2013;8: e80302. doi:10.1371/journal.pone.0080302

17. World Health Organization. Global tuberculosis report 2013. 2013. Available: https://www.who.int/publications/i/item/9789241564656

18. Pefura-Yone EW, Kengne AP, Tagne-Kamdem PE, Afane-Ze E. Clinical significance of low forced expiratory flow between 25% and 75% of vital capacity following treated pulmonary tuberculosis: a cross-sectional study. BMJ Open. 2014;4: e005361. doi:10.1136/bmjopen-2014-005361

19. Pasipanodya JG, Miller TL, Vecino M, Munguia G, Garmon R, Bae S, et al. Pulmonary impairment after tuberculosis. Chest. 2007;131: 1817–1824. doi:10.1378/chest.06-2949

20. Meghji J, Gregorius S, Madan J, Chitimbe F, Thomson R, Rylance J, et al. The long term effect of pulmonary tuberculosis on income and employment in a low income, urban setting. Thorax. 2021;76: 387–395. doi:10.1136/thoraxjnl-2020-215338

21. Khosa C, Bhatt N, Massango I, Azam K, Saathoff E, Bakuli A, et al. Development of chronic lung impairment in Mozambican TB patients and associated risks. BMC Pulm Med. 2020;20: 127. doi:10.1186/s12890-020-1167-1

22. Nishi MP, Mancuzo EV, Sulmonett N, Almeida IN de, César ALA, Miranda SS de. Pulmonary functional assessment: longitudinal study after treatment of pulmonary tuberculosis. Rev Inst Med Trop Sao Paulo. 2021;63: e65. doi:10.1590/S1678-9946202163065

23. Wolbers M, Babiker A, Sabin C, Young J, Dorrucci M, Chêne G, et al. Pretreatment CD4 cell slope and progression to AIDS or death in HIV-infected patients initiating antiretroviral therapy--the CASCADE collaboration: a collaboration of 23 cohort studies. PLoS Med. 2010;7: e1000239. doi:10.1371/journal.pmed.1000239

24. Zhu J, Lyatuu G, Sudfeld CR, Kiravu A, Sando D, Machumi L, et al. Re-evaluating the health impact and cost-effectiveness of tuberculosis preventive treatment for modern HIV cohorts on antiretroviral therapy: a modelling analysis using data from Tanzania. Lancet Glob Health. 2022;10: e1646–e1654. doi:10.1016/S2214-109X(22)00372-2

25. Geng EH, Odeny TA, Lyamuya R, Nakiwogga-Muwanga A, Diero L, Bwana M, et al. Retention in Care and Patient-Reported Reasons for Undocumented Transfer or Stopping Care Among HIV-Infected Patients on Antiretroviral Therapy in Eastern Africa: Application of a Sampling-Based Approach. Clin Infect Dis. 2016;62: 935–944. doi:10.1093/cid/civ1004

26. Vos T, Lim SS, Abbafati C, Abbas KM, Abbasi M, Abbasifard M, et al. Global burden of 369 diseases and injuries in 204 countries and territories, 1990–2019: a systematic analysis for the Global Burden of Disease Study 2019. The Lancet. 2020;396: 1204–1222. doi:10.1016/S0140-6736(20)30925-9

27. Soriano JB, Abajobir AA, Abate KH, Abera SF, Agrawal A, Ahmed MB, et al. Global, regional, and national deaths, prevalence, disability-adjusted life years, and years lived with disability for chronic obstructive pulmonary disease and asthma, 1990–2015: a systematic analysis for the Global Burden of Disease Study 2015. The Lancet Respiratory Medicine. 2017;5: 691–706. doi:10.1016/S2213-2600(17)30293-X

28. Salomon JA, Haagsma JA, Davis A, de Noordhout CM, Polinder S, Havelaar AH, et al. Disability weights for the Global Burden of Disease 2013 study. Lancet Glob Health. 2015;3: e712-723. doi:10.1016/S2214-109X(15)00069-8

29. Wilkinson T, Sculpher MJ, Claxton K, Revill P, Briggs A, Cairns JA, et al. The International Decision Support Initiative Reference Case for Economic Evaluation: An Aid to Thought. Value Health. 2016;19: 921–928. doi:10.1016/j.jval.2016.04.015

30. Stenberg K, Lauer JA, Gkountouras G, Fitzpatrick C, Stanciole A. Econometric estimation of WHO-CHOICE country-specific costs for inpatient and outpatient health service delivery. Cost Effectiveness and Resource Allocation. 2018;16: 11. doi:10.1186/s12962-018-0095-x

31. Siapka M, Vassall A, Cunnama L, Pineda C, Cerecero D, Sweeney S, et al. Cost of tuberculosis treatment in low- and middle-income countries: systematic review and meta-regression. Int J Tuberc Lung Dis. 2020;24: 802–810. doi:10.5588/ijtld.19.0694

32. Opuni M, Sanchez-Morales JE, Figueroa JL, Salas-Ortiz A, Banda LM, Olawo A, et al. Estimating the cost of HIV services for key populations provided by the LINKAGES program in Kenya and Malawi. BMC Health Services Research. 2023;23: 337. doi:10.1186/s12913-023-09279-w

33. Portnoy A, Yamanaka T, Nguhiu P, Nishikiori N, Garcia Baena I, Floyd K, et al. Costs incurred by people receiving tuberculosis treatment in low-income and middle-income countries: a meta-regression analysis. Lancet Glob Health. 2023;11: e1640–e1647. doi:10.1016/S2214-109X(23)00369-8

34. Malawi National TB and Leprosy Eliminzation Programme. Tuberculosis Active Case Finding Report 2020. 2020.

35. World Health Organization. WHO consolidated guidelines on tuberculosis: Module 2: screening – systematic screening for tuberculosis disease. Geneva: World Health Organization; 2021. Available: http://www.ncbi.nlm.nih.gov/books/NBK569338/

36. Zifodya JS, Kreniske JS, Schiller I, Kohli M, Dendukuri N, Schumacher SG, et al. Xpert Ultra versus Xpert MTB/RIF for pulmonary tuberculosis and rifampicin resistance in adults with presumptive pulmonary tuberculosis. Cochrane Database Syst Rev. 2021;2: CD009593. doi:10.1002/14651858.CD009593.pub5

37. Veeken LD, Schwalb A, Horton KC, Koesoemadinata RC, Alisjahbana B, van Crevel R, et al. Mind the clinic-community gap: how concerned should we be about false positive test results in mass tuberculosis screening. J Infect Dis. 2025; jiaf268. doi:10.1093/infdis/jiaf268
