# Supplementary material for: Potential health impacts and costs of active case finding guided by Mycobacterium tuberculosis immunoreactivity survey results in Blantyre, Malawi: A mathematical modeling study
Source: PLoS Med. 2026 Oct 7;23(10):e1005272. doi: 10.1371/journal.pmed.1005272 (PMC13657404; doi:10.1371/journal.pmed.1005272)
Supplement: Supplementary file 3 [file pmed.1005272.s003.docx]

**Potential health impacts and costs of active case finding guided by Mycobacterium tuberculosis immunoreactivity survey results in Blantyre, Malawi (PMEDICINE-D-25-03673R1)**

**Response to reviewers**

**Reviewer #1:**

1. The study is clearly and thoroughly analysed and presented. However, I have a number of questions that do not appear to be covered in the description of the intervention and measurement of outcomes (effectiveness).

**Response**: Thank you for this assessment. We have edited the text to add the requested details, as described in comments below.

2. What is the justification for limiting the ACF to one round of screening? Both empirical and modelling studies of ACF have shown that benefits are cumulative and repeated screening is required to demonstrate effectiveness on prevalence, transmission and incidence, and hence on health outcomes (mortality and DALYs).

**Response**: We chose to model one round of ACF screening as a proof of concept, as the first round of ACF is when the individual health benefits and cost-effectiveness will be greatest. We agree that results for repeated ACF would also be of interest to some readers and would likely produce additional health benefits. We have added text reflecting these points to the Limitations section:

“Fifth, in this study we examined the impact and cost-effectiveness of a single round of ACF. However, in some settings, ACF may be implemented as repeated rounds, which could generate additional health benefits by identifying incident cases over time. However, subsequent rounds are likely to yield diminishing returns as the pool of undiagnosed prevalent cases declines while program costs remain substantial [49]. We did not explicitly model repeated rounds, and the incremental costs and benefits of such strategies may differ from those presented here.” [lines 422-428]

3. Does the model incorporate the effect of the intervention on transmission and subsequent incidence (which is additional to the benefit for the individuals who are diagnosed by the intervention). Without including these compounding benefits, the effectiveness is substantially underestimated and hence ICERs are substantially over-estimated.

**Response**: The reviewer is correct; in the original analysis we did not include the consequences of ACF for transmission and secondary TB cases. We agree that this approach will lead to underestimation of effectiveness and cost-effectiveness. Based on the Reviewer’s suggestion, we have now revised our modelling approach to incorporate the additional benefits associated with averted secondary TB cases. For the revised **main analysis** we have now estimated the number of **1^st^-generation secondary TB cases averted** and incorporated the health benefits and cost-savings associated with these averted TB cases when estimating effectiveness and cost-effectiveness. In sensitivity analyses we present results for the **original analysis (no transmission effects)**, as well as an analysis that considers **all potential subsequent generations of TB cases averted**. Text edits associated with this change are shown below. Based on this revision (and other smaller analytic revisions described below), the ICER for targeted ACF vs. no ACF changed from $1,199 per DALY averted (no transmission effects, Table M in S1 Appendix) to $682 per DALY averted (accounting for 1st generation transmission effects, Table 3), assuming P (ARTI predictive power) = 1.0.

*Methods section text*

“The incremental health benefits of ACF were calculated based on the consequences for individuals receiving ACF (benefits for individuals receiving true-positive diagnoses and harms for individuals receiving false-positive diagnoses) as well as health benefits resulting from averted secondary TB cases. Incremental costs for ACF scenarios were calculated from the direct costs of the ACF intervention (assumed to cost US$7 per person receiving ACF) and associated TB treatment, cost-savings from averted TB diagnosis and treatment through passive diagnosis, cost-savings resulting from averted secondary cases, and changes in ART costs due to changes in survival among individuals with HIV.” [lines 132-139]

“*Approach for estimating secondary TB cases averted by ACF*

We estimated the number of secondary TB cases averted based on the numbers of individuals with TB diagnosed and treated through ACF and the reproductive number for Blantyre. The reproductive number was estimated from the secular trend in TB incidence (assumed to be proportional to the TB notification rate), and published estimates of the generation time [25–27]. For individuals with TB identified through ACF we assumed transmission would be reduced by 50% as compared to the PCF scenario. For each averted TB case, incremental health benefits and costs were based on the health and economic outcomes for individuals with vs. without TB, calculated using the Markov model. In the main analysis we considered costs and health benefits associated with the first generation of secondary TB cases averted by ACF. In sensitivity analyses, we recalculated costs and health consequences considering all future secondary cases, and conducted an additional analysis excluding transmission benefits.” [lines 184-194]

*Results section text*

“*Secondary TB cases averted by ACF*

With ACF, the reduction in transmission resulting from earlier TB identification and treatment through ACF was estimated to avert 0.30 (95% CrI: 0.13–0.51) first-generation secondary TB cases per index case. For each of these averted TB cases we estimated there would be 8.38 (95% CrI: 5.56–11.86) life-years gained and 14.79 (95% CrI: 9.84–20.81) DALYs averted. Accounting for the estimated number of secondary cases averted and the health burden associated with each case, each TB case identified through ACF was estimated to yield an additional 1.73 (95% CrI: 0.70–3.27) life-years gained and 3.10 (95% CrI: 1.20–5.75) DALYs averted through reduced transmission.” [lines 273-280]

*Discussion section text*

“Second, we computed the number of TB cases that could be averted by ACF based on an estimate of the reproductive number for Blantyre and an assumption that early diagnosis via ACF would avert approximately half of the transmission associated with each TB case. However, there is substantial uncertainty about the effects of ACF on transmission, and sensitivity analyses that tested optimistic and pessimistic transmission assumptions found that cost-effectiveness ratios were sensitive to the magnitude of transmission effects.” [lines 409-415]

4. Related to the above, what is the time horizon for measuring the outcomes?

**Response**: The time horizon for the analysis was the lifetime of individuals developing (or not developing) TB. We have edited the text to clarify this.

“All costs and health outcomes were calculated over the lifetime of affected individuals.” [lines 139-140]

5. Results are cited as " improve life expectancy by ….  for individuals with TB disease". This is not a relevant outcome. The relevant outcome is improved life expectancy (and reduction in DALYs) for the population as a whole and this includes the effective on incidence of TB prevented over a long time horizon due to reduced transmission (due to reduced prevalence).

**Response**: We agree with the reviewer that including the number of averted secondary cases improves the analysis. In the revised analyses, described above, we have now included these outcomes.

6. What are the assumptions about participation rates in screening and rates of successful transition for TB diagnosis to completing TB treatment (in all three arms)?

**Response**: In the analysis these parameters were informed by consultation and review of data with the Malawi National TB and Leprosy Elimination Programme, as well as published literature. We assumed that (a) all residents are offered ACF (for the two ACF arms), (b) 10.2% of those offered ACF refuse, or are unable to provide sputum, (c) 5.0% of those diagnosed with TB (through ACF or PCF) are lost to follow-up before treatment initiation and (d) individuals initiating treatment experience treatment outcomes (death, loss to follow-up, failure, and cure) consistent with reported treatment outcomes data. We have clarified these points in the manuscript.

“Under the ACF scenarios we assumed that all residents in a given cluster would be offered ACF, 10.2% of those offered ACF would refuse or be unable to provide sputum [28], 5.0% of those diagnosed with TB would be lost to follow-up before treatment initiation (based on NTLEP input), and individuals initiating treatment would experience treatment outcomes (death, loss to follow-up, failure, and cure) consistent with reported treatment outcomes data [29].” [lines 200-205]

7. My other comment would be that the cost of screening using the proposed algorithm may be over-estimated based on newer developments. I realise this may not need to be estimated but it should be acknowledged that ICER would be lower if the cost of screening could be lowered, based on evolution of new tools, without losing effectiveness.

**Response**: We have added this point to the limitations paragraph of the discussion.

“Fourth, we only examined one ACF diagnostic algorithm, yet different approaches have been proposed [48], and new technologies may soon be available that lower the cost of testing.” [lines 420-422]

**Reviewer #2:**

8. In their manuscript "Potential health impacts and costs of active case finding guided by Mycobacterium tuberculosis immunoreactivity survey results in Blantyre, Malawi", Kim et al. present a mathematical model assessing the effect and cost-effectiveness of a tuberculosis (TB) active case finding (ACF) intervention. In the model, the geographical implementation of the ACF intervention is guided by TB immunoreactivity survey results in children <5 years, serving as a surrogate parameter for the annual risk of TB infection. Overall, I find the study to be very well conducted, with methods presented clearly, valuable results, and a sound introduction and discussion. Below, I offer a few comments the authors might want to consider to further improve the manuscript.

**Response**: Thank you for these kind words.

9. Methods: On page 4, the authors describe the setting the model is parameterized to. Given that Blantyre, Malawi is a setting with a high TB and HIV burden, the generalisability of the model's results to other epidemiological settings is limited. Thus, perhaps as a sensitivity analysis, I would suggest to assess how the model's results change in settings with a lower TB and HIV burden.

**Response**: We agree that results would differ in settings with higher or lower HIV or TB burden. As we have undertaken this study specifically for Blantyre, we believe it would require a substantial extension of the study to represent these other settings, particularly where HIV is less common. Moreover, a substantial number of individuals in eastern and southern Africa live in settings with co-prevalent HIV, for which these results may provide some guidance. We have edited the Discussion section to note the sensitivity of results to TB and HIV prevalence.

“Lastly, this study was conducted in a setting of co-prevalent HIV and TB, and the health impact and cost-effectiveness of ACF will vary according to the prevalence of these conditions.” [lines 428-430]

10. On page 5, I found a detailed description of the ACF intervention, but only very limited information about the passive case-finding (PCF) only scenario. I would suggest to add a brief description of the PCF scenario, including the tool to diagnose TB, linkage to treatment, and whether treatment outcomes differ for individuals detected through PCF versus ACF.

**Response**: We have edited the text to provide additional details of the PCF scenario.

“PCF was assumed to occur through the routine health system, with symptomatic clinic attendees receiving clinical evaluation and bacteriological testing through the Malawi Ministry of Health primary and secondary healthcare systems. As compared to ACF, individuals identified through PCF were assumed to face additional risks of mortality before and during treatment, and elevated rates of post-TB sequelae following treatment completion.” [lines 118-122]

11. On page 6, 2022 USD are defined as the currency used in the model. Is there a reason 2022 USD are used instead of 2025 USD? I would suggest that the authors either state this reason or update costs to 2025 USD.

**Response**: Thank you for this suggestion. We have updated the cost year to 2024, as this is the most recent year for which price inflation data are available (annual GDP deflator reported by the World Bank).

12. On page 7, a 5% pre-treatment loss to follow-up is described as part of the ACF intervention. However, I could not find any description of loss to follow-up due to inability to expectorate sufficient sputum for Xpert testing, even though the proportion of individuals not able to produce sputum can be substantial (see e.g., [https://doi.org/10.1101/2025.11.02.25339326)](https://urldefense.proofpoint.com/v2/url?u=https-3A__doi.org_10.1101_2025.11.02.25339326-29&d=DwMGaQ&c=WO-RGvefibhHBZq3fL85hQ&r=ZqDaSE5Uw14qWqZKqjbbB7wi83lTNnDiYqN7D_rgR5A&m=AIj6f9mq8kPhRywyrch6PLh4eh2aNFzedD7hTti9T1n6_wqaqeD6cjBxOAXbJ5JA&s=aCfYYo4yHcGPc8IZx0fs0-Bw9RBI21OULmiQUb6mSsY&e=). It would be very helpful if the authors could clarify whether the possibility of insufficient sputum expectoration is considered in the model. In case the possibility of insufficient sputum expectoration is not yet included in the model, I would to incorporate it.

**Response**: Thank you for raising this. In the original analysis we did not consider the possibility of inability to produce sputum. We have now incorporated this into the analysis based on estimates from a trial of community-based screening (<https://doi.org/10.5588/ijtldopen.24.0319>), in which 7.4% could not produce sputum and 2.8% declined to provide sputum (total 10.2%).

“Under the ACF scenarios we assumed that all residents in a given cluster would be offered ACF, 10.2% of those offered ACF would refuse or be unable to provide sputum [28], 5.0% of those diagnosed with TB would be lost to follow-up before treatment initiation (based on NTLEP input), and individuals initiating treatment would experience treatment outcomes (death, loss to follow-up, failure, and cure) consistent with reported treatment outcomes data [29].” [lines 200-205]

13. Results: On page 10, in the last sentence of the first paragraph, I would suggest to state the exact average decrease in life-expectancy through unnecessary TB treatment instead of stating 'limited but non-negligible impact'.

**Response**: We have added this detail:

“False-positive results among individuals without TB, leading to unnecessary TB treatment, were associated with a small decrease in life expectancy of 3.2e⁻⁵ years (95% CrI: 3.9e⁻⁶–7.0e⁻⁵) among HIV-negative individuals and 1.4e⁻⁵ years (95% CrI: 0–4.3e⁻⁵) among people living with HIV.” [lines 260-263]

14. On page 21, table 2, please specify why the bottom six ICERs are marked in grey - in the appendix, only clusters where targeted ACF is dominated are marked in grey.

**Response**: We apologize for the confusion. As the reviewer correctly noted, only clusters where targeted ACF is dominated were intended to be marked in grey. The previous shading in Table 2 was an error, which we have now corrected this accordingly.

15. Discussion: On page 13/14, in the paragraph discussing the availability of Mtb immunoreactivity survey data, it would be helpful if the authors could include a few examples where such data for children <5 years is readily available.

**Response**: We have edited this section to discuss current availability of these data, and considerations for further use of these surveys.

“Historically, ARTI data have been widely used to track TB epidemiology and inform public health policy and planning, including prioritization of interventions such as case finding, preventive therapy, and vaccination strategies [38–42]. These surveys have been conducted less frequently in recent years, with greater priority being given to surveys of TB disease prevalence for tracking changes in TB epidemiology. However, compared to TB prevalence surveys immunoreactivity surveys are considerably less resource intensive to implement, and can be feasibly integrated into existing surveillance systems, particularly within primary care settings [43]. For these reasons, there have been calls to expand the use of immunoreactivity surveys to supplement other methods for tracking TB burden.” [lines 360-368]

16. On page 14, in the last paragraph, the authors argue that their findings of ACF potentially mitigating excess TB-associated mortality contrasts the findings of the TREATS study, which did not find a relevant epidemiological effect of a combined community-based TB/HIV testing and care intervention. They mention the exclusion of dynamic transmission effects in their model as one of the potential reasons for this difference. To me, this reads as if the difference in results would have been smaller had dynamic transmission effects been included in the present model. Is this what the authors meant to say? Shouldn't the inclusion of dynamic transmission effects lead to an even larger benefit of ACF, thus further increasing in the difference in results of the present model and the results of the TREATS study?

**Response**: Thank you for catching this error, and we agree with the reviewer on this point. Moreover, we have now revised our analysis to include transmission effects. We have revised this section accordingly.

“However, there are several differences between our study and the TREATS model, including differences in the approach for screening (TREATS primarily relied on symptom screening, whereas our ACF strategy also used chest X-ray with computer-aided detection for individuals without symptoms), and targeting (our ARTI-guided approach allowed fine-scale targeted of high-transmission neighborhoods, with potentially greater impact on transmission).” [lines 385-390]

**Reviewer #3:**

17. Thank you to the authors for submitting this excellent paper, I very much enjoyed reading it. It is on a clearly important topic, very well written and has a nice balanced discussion of the results. I have a few minor comments that I think the authors may want to look at before publication can be considered.

**Response**: Thank you for these kind words.

18. I think Appendix Figure 1 needs to be moved into the main text.

**Response**: We agree that this figure would be helpful for some readers and have moved this to the main text (now Figure 1).

19. We need much more information about the Markov model. This is should be in the main text rather than the appendices. Can't assume that the reader will have read the Can et al (2025) paper.

**Response**: We have added substantial additional text describing the Markov model to the Methods section.

“We used a published Markov simulation model [21] to estimate lifetime health outcomes and TB care costs for individuals with or without TB and/or HIV under each intervention scenario. Using this model a cohort of 10,000 individuals representing the ACF target population was exposed to different TB diagnosis and treatment scenarios and tracked over time to estimate cumulative health outcomes and costs. Individuals were represented in the model by starting age (mean: 34.0, IQR: 26.0–40.0), sex (75.0% male for individuals with TB), HIV and ART status (13.6% HIV-positive, 70.1% of these on ART), CD4 cell count for individuals with HIV (mean: 274 cells/μL, IQR: 68–372 cells/μL), and FEV1 percentage as a measure of TB-associated lung damage (mean: 63%, IQR: 52–75% for individuals with prevalent TB). **Figure A** in **S1 Appendix** provides a schematic of the model structure. In the model, individual survival and health status were updated with a weekly timestep, until each individual in the simulated cohort was dead. HIV-associated immune function was tracked through changes in CD4 cell count, which declined for individuals not receiving ART, and rebounded among individuals receiving ART. Individuals with HIV had greater HIV-associated mortality and disability weights, determined by CD4 cell count and ART receipt. HIV-associated immune function also affected rates of TB progression and probability of self-cure. For individuals with untreated TB, those receiving ART were assumed to have higher rates of diagnostic evaluation compared to individuals not receiving ART. Lung function was tracked through changes in FEV1 percentage, which was assumed to decline for individuals with untreated TB disease, rebound during TB treatment, and continue to improve following successful TB treatment. The extent of lung function recovery following TB cure was determined by the minimum FEV1 value reached before treatment initiation. FEV1 percentage was assumed to continue to decline for individuals receiving a false-negative TB diagnosis, lost to follow-up from treatment before TB cure, or with ongoing disease following treatment completion. TB-associated mortality and disability were determined by TB status and FEV1 percentage. Individuals surviving TB disease were assumed to face ongoing morbidity and mortality risks due to post-TB sequelae. The magnitude of these risks was based on the extent of residual long impairment as measured by FEV1 percentage. As compared to the earlier application of the model [21], this analysis adopted higher values for the FEV1 distribution for individuals with TB, reflecting a milder distribution of TB disease compared to individuals detected through passive case detection. Key model parameters are provided in **Table A** in **S1 Appendix**, and the model was programmed in R and C++. Additional details are reported in Can et al., 2025 [21].” [lines 144-175]

20. I think we need the information from Appendix Table 1 in the main text, either as an actual table or described in the text.

**Response**: These details have been moved to the main text.

“Individuals were represented in the model by starting age (mean: 34.0, IQR: 26.0–40.0), sex (75.0% male for individuals with TB), HIV and ART status (13.6% HIV-positive, 70.1% of these on ART), CD4 cell count for individuals with HIV (mean: 274 cells/μL, IQR: 68–372 cells/μL), and FEV1 percentage as a measure of TB-associated lung damage (mean: 63%, IQR: 52–75% for individuals with prevalent TB). [lines 148-152]

21. I think we need a little more information regarding the key costs in the main text too. Not all the costs from Appendix Table 2, but some of the key ones described in the text to get a feel for the type of level of costs we're dealing with.

**Response**: We thank the reviewer for this helpful suggestion. We have now included additional information in the main text summarizing key cost inputs to provide readers with a clearer sense of the magnitude and composition of costs, while retaining full details in Appendix Table B.

“Incremental costs for ACF scenarios were calculated from the direct costs of the ACF intervention (assumed to cost US$7 per person receiving ACF) and associated TB treatment, cost-savings from averted TB diagnosis and treatment through passive diagnosis, cost-savings resulting from averted secondary cases, and changes in ART costs due to changes in survival among individuals with HIV. All costs and health outcomes were calculated over the lifetime of affected individuals.” [lines 135-140]

22. I think you need to be a little clearer regarding the different intervention scenarios in the model. In the 'Intervention Scenarios' section you talk about PCF Only, Untargeted ACF and Targeted ACF, whereas the results only report ACF vs no ACF. It's a little confusing.

**Response**: Thank you for this comment, we have revised the references to these scenarios in the results section for clarity.

23. I'm a little confused where the 48% figure came when assessing the the implications of geographical targeting. Is just because it was roughly half and fitted the clusters? One sentence regarding this would be helpful.

**Response**: Thank you for raising this point. We have revised the analysis and text so that geographical targeting was implemented to reach exactly 50% population coverage by ranking clusters and including clusters sequentially until the target was reached. For the boundary cluster, a proportional inclusion was applied to achieve 50% coverage precisely. The manuscript has been updated to describe this approach.

“To further assess the implications of geographic targeting, we modelled a scenario in which ACF was implemented in clusters comprising half of the total population, starting with clusters with the highest ARTI. Clusters were ranked and included sequentially until reaching 50% population coverage.” [lines 229-233]

24. In the discussion you state that unlike the TREATS model you didn't include dynamic transmission effects in your model. I was wondering if the authors could comment on why they didn't include this in their model, how much of a disadvantage this is and how it may impact the results of their study.

**Response**: The reviewer is correct that in the original analysis we did not include the consequences of ACF for transmission. Excluding these transmission effects could under-estimate the health impact of the intervention. For this reason, we have now revised our modelling approach to incorporate the additional benefits associated with averted secondary TB cases, as well as presenting alternative approaches for quantifying these transmission effects in sensitivity analyses.

25. Is there a better way of presenting the results in Table 1, Appendix Table 5 and Appendix Table 6? It looks really odd and a bit messy having different numbers of decimal places in different rows of the table

**Response**: We have revised the presentation of values in these Tables.

**Reviewer #4:**

26. Kim and colleagues present their analysis of the impact of ACf vs PCF in Malawi, for a strategy of targeted intervention guided to specific districts in Blantyre using survey data from Mtb immunoreactivity under 5 years olds.

**Response**: Thank you for this assessment.

27. Major Comments
    Lack of detail regarding the underlying model structure and inputs
    The analysis relies heavily on a Markov microsimulation model described as being "developed by Can et al. (2025)" with only a brief reference stating "The model incorporated transitions between health states, including undiagnosed TB, diagnosis and treatment, post-TB sequelae, and death" (page 6, Methods). This creates a significant transparency issue as readers must consult an external publication to understand fundamental aspects of the analysis, like the complete model structure and health states, transition probabilities between states, natural history assumptions, how HIV status affects transitions, age stratification and demographic processes. In light of this, the Methods section should be restructured to first provide a clear, self-contained description of the model architecture before explaining how it was applied to this specific question. Consider adding a diagram showing the complete model structure with all health states and transitions, a table of key transition parameters, clear statement that this analysis applies a previously published and validated model to a new question (ARTI-guided targeting) and an explicit description of which aspects are novel to this study versus which are from the prior work.

**Response**: Thank you for this suggestion. We have substantially expanded the description of the model in the Methods and also added a parameter table and model schematic to the appendix.

“We used a published Markov simulation model [21] to estimate lifetime health outcomes and TB care costs for individuals with or without TB and/or HIV under each intervention scenario. Using this model a cohort of 10,000 individuals representing the ACF target population was exposed to different TB diagnosis and treatment scenarios and tracked over time to estimate cumulative health outcomes and costs. Individuals were represented in the model by starting age (mean: 34.0, IQR: 26.0–40.0), sex (75.0% male for individuals with TB), HIV and ART status (13.6% HIV-positive, 70.1% of these on ART), CD4 cell count for individuals with HIV (mean: 274 cells/μL, IQR: 68–372 cells/μL), and FEV1 percentage as a measure of TB-associated lung damage (mean: 63%, IQR: 52–75% for individuals with prevalent TB). **Figure A** in **S1 Appendix** provides a schematic of the model structure. In the model, individual survival and health status were updated with a weekly timestep, until each individual in the simulated cohort was dead. HIV-associated immune function was tracked through changes in CD4 cell count, which declined for individuals not receiving ART, and rebounded among individuals receiving ART. Individuals with HIV had greater HIV-associated mortality and disability weights, determined by CD4 cell count and ART receipt. HIV-associated immune function also affected rates of TB progression and probability of self-cure. For individuals with untreated TB, those receiving ART were assumed to have higher rates of diagnostic evaluation compared to individuals not receiving ART. Lung function was tracked through changes in FEV1 percentage, which was assumed to decline for individuals with untreated TB disease, rebound during TB treatment, and continue to improve following successful TB treatment. The extent of lung function recovery following TB cure was determined by the minimum FEV1 value reached before treatment initiation. FEV1 percentage was assumed to continue to decline for individuals receiving a false-negative TB diagnosis, lost to follow-up from treatment before TB cure, or with ongoing disease following treatment completion. TB-associated mortality and disability were determined by TB status and FEV1 percentage. Individuals surviving TB disease were assumed to face ongoing morbidity and mortality risks due to post-TB sequelae. The magnitude of these risks was based on the extent of residual long impairment as measured by FEV1 percentage. As compared to the earlier application of the model [21], this analysis adopted higher values for the FEV1 distribution for individuals with TB, reflecting a milder distribution of TB disease compared to individuals detected through passive case detection. Key model parameters are provided in **Table A** in **S1 Appendix**, and the model was programmed in R and C++. Additional details are reported in Can et al., 2025 [21].” [lines 144-175]

28. Post-TB sequelae modeling
    The manuscript mentions that the model includes "post-TB sequelae" as a health state and notes these "are increasingly recognized as major contributors to TB disease burden" (page 14, Discussion). The model appears to account for post-TB morbidity through YLDs and ongoing productivity losses (Appendix Table 2). However, critical details are missing, namely: What specific post-TB sequelae are included (respiratory impairment, cardiovascular complications, etc.)? What proportion of treated/untreated TB cases progress to sequelae? How do sequelae severity and duration differ by HIV status? What disability weights are applied? How do post-TB sequelae affect mortality risk? Given that post-TB sequelae appear to influence the cost-effectiveness estimates (through ongoing costs and DALYs), these assumptions require transparent reporting. Perhaps add a dedicated subsection or table describing the post-TB sequelae model component, including transition probabilities to sequelae states, disability weights, mortality risks, and costs associated with this state. If these details are only in Can et al. (2025), they should be summarized here given their importance to the current analysis.

**Response**: These details have been incorporated into the new Methods section text described above, with parameter values included in the parameter table (**Table A** in **S1 Appendix**).

29. MTB immunoreactivity surveys as a targeting tool
    While the novelty of using Mtb immunoreactivity surveys in children <5 years for ACF targeting is central to this work, there is insufficient critical discussion of this approach's strengths and limitations. The manuscript briefly mentions that "immunoreactivity surveys are considerably less intensive than TB prevalence surveys" (page 14) and that the survey used convenience sampling from primary care, but several important issues receive inadequate attention, like feasibility and acceptability of testing young children; selection bias from convenience sampling (children attending clinics may not represent community burden); Uncertainty in ARTI estimates, particularly in clusters with zero positives (acknowledged briefly but not discussed in depth); The assumption that infection risk in children <5 reflects recent transmission and correlates with prevalent disease (which is central but examined only through sensitivity analysis). The manuscript acknowledges in limitations that "the strength of this relationship [between ARTI and TB prevalence] may vary" but doesn't adequately discuss the implications of the convenience sampling approach or situate this method among alternatives.

**Response**: We have revised and extended this paragraph to discuss these issues.

“While providing potentially valuable data, *Mtb* immunoreactivity surveys can introduce a number of challenges, including the feasibility and acceptability of testing young children, which were not examined in this analysis. Moreover, multiple factors could weaken the relationship between immunoreactivity survey estimates and true TB prevalence, including statistical uncertainty from small sample sizes, differential sampling bias across clusters, imperfect test performance, and uncertainty in the quantitative relationship between immunoreactivity among children and population TB prevalence. We tested the implications of these factors through varying the predictive power of ARTI, but more research on this relationship is needed. [lines 371-379]

30. Additional Comments
    Minor point on model description: The Methods would benefit from explicitly stating upfront (perhaps in the first paragraph of "Modelling approach") that the analysis applies a previously developed and validated TB natural history model to assess a novel intervention targeting strategy. Currently, this only becomes clear midway through the Methods section.

**Response**: The use of an existing model is described in the first sentence of this section.

“We used a published Markov simulation model [21] to estimate lifetime health outcomes and TB care costs for individuals with or without TB and/or HIV under each intervention scenario.” [lines 144-145]

31. These comments aim to help the authors improve transparency and strengthen the manuscript's contribution. The work addresses an important question, but clearer presentation of the underlying model and more critical engagement with the surveillance approach would substantially enhance its value to the field.

**Response**: We thank the reviewer for their comments. We hope that the edits and additions described above have improved the presentation of this work.

**Reviewer #5:**

32. Sun and colleagues use mathematical models to evaluate the effectiveness and cost-effectiveness of active TB case finding in specific areas of Blantyre, Malawi, either in a universal manner, or targeted to higher risk areas based on estimated annual risk of TB infection. They conclude that when compared to passive case finding, any active approach prevents TB and DALYs, but that a targeted approach is more efficient (cost-effective) than a universal approach. The authors present an intriguing way to think about how to target case finding, highlighting the use of TB infection surveys as a more inexpensive alternative to identifying higher risk areas than activities like prevalence surveys.

**Response**: Thank you for these comments.

MAJOR

33. The authors do not include the costs of the initial TB infection prevalence survey that was used to inform the targeting of case finding. While in this specific circumstance the survey was already performed, this will not always be the case. The authors acknowledge this will add cost, but this is a potentially important cost and would be important to include as it would be necessary for implementation elsewhere. Costs of this survey could be annuitized over the expected timeframe from when such a survey would need to be repeated, as if indeed active case finding was guided by such surveys, their impact on epidemiology would need to be monitored so they could be continuously re-targeted.

**Response**: We agree this is an important point. While we were unable to incorporate these costs in this analysis, we have expanded the relevant section of the Discussion raising the points made.

“The cost of collecting immunoreactivity survey data is a factor that could be influential for cost-effectiveness, and so while excluded from this analysis these costs would need to be considered in other settings where these data are not available. For this reason, research is needed on how to best conduct these surveys, how to make them more efficient, and the frequency with which they need to be repeated.” [lines 416-420]

34. The authors do not consider the impacts of their intervention on transmission. This is another acknowledged limitation, but an important one, particularly in a high-burden setting with an epidemic driven by HIV. In some of the clusters, estimated annual risk of infection was as high as 8%, suggesting high-levels of ongoing transmission. I do not think the inclusion of transmission would necessarily reduce the estimated ICERs to local willingness-to-pay thresholds, but I do think it may have an important impact, particularly in these estimated-to-be high transmission areas.

**Response**: Thank you for this point. We have now adjusted the analysis to incorporate the additional benefits associated with averted secondary TB cases. For the main analysis we estimated the number of **1^st^-generation secondary TB cases averted**, and incorporated the health benefits and cost-savings associated with these averted TB cases when estimating effectiveness and cost-effectiveness. In sensitivity analyses we present results for the **original analysis (no transmission effects)**, and an analysis that considers **all potential generations of TB cases**. Text edits associated with this change are shown below. Based on this revision (and other smaller analytic revisions described below), the ICER for targeted ACF vs. no ACF changed from $1,199 per DALY averted (no transmission effects, Table M in S1 Appendix) to $682 per DALY averted (accounting for 1st generation transmission effects, Table 3), assuming P (ARTI predictive power) = 1.

*Methods section text*

“The incremental health benefits of ACF were calculated based on the consequences for individuals receiving ACF (benefits for individuals receiving true-positive diagnoses and harms for individuals receiving false-positive diagnoses) as well as health benefits resulting from averted secondary TB cases. Incremental costs for ACF scenarios were calculated from the direct costs of the ACF intervention (assumed to cost US$7 per person receiving ACF) and associated TB treatment, cost-savings from averted TB diagnosis and treatment through passive diagnosis, cost-savings resulting from averted secondary cases, and changes in ART costs due to changes in survival among individuals with HIV.” [lines 132-139]

“*Approach for estimating secondary TB cases averted by ACF*

We estimated the number of secondary TB cases averted based on the numbers of individuals with TB diagnosed and treated through ACF and the reproductive number for Blantyre. The reproductive number was estimated from the secular trend in TB incidence (assumed to be proportional to the TB notification rate), and published estimates of the generation time [25–27]. For individuals with TB identified through ACF we assumed transmission would be reduced by 50% as compared to the PCF scenario. For each averted TB case, incremental health benefits and costs were based on the health and economic outcomes for individuals with vs. without TB, calculated using the Markov model. In the main analysis we considered costs and health benefits associated with the first generation of secondary TB cases averted by ACF. In sensitivity analyses, we recalculated costs and health consequences considering all future secondary cases, and conducted an additional analysis excluding transmission benefits.” [lines 184-194]

*Results section text*

“*Secondary TB cases averted by ACF*

With ACF, the reduction in transmission resulting from earlier TB identification and treatment through ACF was estimated to avert 0.30 (95% CrI: 0.13–0.51) first-generation secondary TB cases per index case. For each of these averted TB cases we estimated there would be 8.38 (95% CrI: 5.56–11.86) life-years gained and 14.79 (95% CrI: 9.84–20.81) DALYs averted. Accounting for the estimated number of secondary cases averted and the health burden associated with each case, each TB case identified through ACF was estimated to yield an additional 1.73 (95% CrI: 0.70–3.27) life-years gained and 3.10 (95% CrI: 1.20–5.75) DALYs averted through reduced transmission.” [lines 273-280]

*Discussion section text*

“Second, we computed the number of TB cases that could be averted by ACF based on an estimate of the reproductive number for Blantyre and an assumption that early diagnosis via ACF would avert approximately half of the transmission associated with each TB case. However, there is substantial uncertainty about the effects of ACF on transmission, and sensitivity analyses that tested optimistic and pessimistic transmission assumptions found that cost-effectiveness ratios were sensitive to the magnitude of transmission effects.” [lines 409-415]

35. MINOR

Do the authors have TB incidence among each of the clusters included in the annual risk of infection analysis? Some clusters had no infection detected during the survey. Knowing the incidence data for these clusters in relation to the estimated annual risk of infection would provide the reader some ability to understand the face validity of the annual risk of infection estimates (i.e., are they reasonable, given the convenience sampling approach used).

**Response**: Unfortunately, we do not have TB incidence data for the clusters. Given suboptimal TB case detection in this setting (as in other similar settings), unbiased incidence data are extremely challenging to obtain. For this reason, we examined how results changed under a range of different assumptions for the predictive power of the ARTI estimates.

36. I understand many model details are published elsewhere, but given no limitations on the length of the supplement, it would be helpful to have the complete list of modeling parameters and approaches included for ease of reference. Given the aim of the study referenced (Can et al) is to look at progressive lung damage and implications of different treatment delays on this outcome, necessary modifications to Blantyre (a setting not included in the original model) and the specific questions evaluated here, should be explained. Can the authors also supply sources for demographic parameters used (e.g., I was surprised to see 75% of the population male).

**Response**: Thank you for this suggestion. We have now added substantial additional model details in the Methods section (quoted below), as well as a table of parameter values and sources in the Supplement (Table A in S1 Appendix). For point on the percentage male, this was for individuals with TB, which we have now specified in the text.

“We used a published Markov simulation model [21] to estimate lifetime health outcomes and TB care costs for individuals with or without TB and/or HIV under each intervention scenario. Using this model a cohort of 10,000 individuals representing the ACF target population was exposed to different TB diagnosis and treatment scenarios and tracked over time to estimate cumulative health outcomes and costs. Individuals were represented in the model by starting age (mean: 34.0, IQR: 26.0–40.0), sex (75.0% male for individuals with TB), HIV and ART status (13.6% HIV-positive, 70.1% of these on ART), CD4 cell count for individuals with HIV (mean: 274 cells/μL, IQR: 68–372 cells/μL), and FEV1 percentage as a measure of TB-associated lung damage (mean: 63%, IQR: 52–75% for individuals with prevalent TB). **Figure A** in **S1 Appendix** provides a schematic of the model structure. In the model, individual survival and health status were updated with a weekly timestep, until each individual in the simulated cohort was dead. HIV-associated immune function was tracked through changes in CD4 cell count, which declined for individuals not receiving ART, and rebounded among individuals receiving ART. Individuals with HIV had greater HIV-associated mortality and disability weights, determined by CD4 cell count and ART receipt. HIV-associated immune function also affected rates of TB progression and probability of self-cure. For individuals with untreated TB, those receiving ART were assumed to have higher rates of diagnostic evaluation compared to individuals not receiving ART. Lung function was tracked through changes in FEV1 percentage, which was assumed to decline for individuals with untreated TB disease, rebound during TB treatment, and continue to improve following successful TB treatment. The extent of lung function recovery following TB cure was determined by the minimum FEV1 value reached before treatment initiation. FEV1 percentage was assumed to continue to decline for individuals receiving a false-negative TB diagnosis, lost to follow-up from treatment before TB cure, or with ongoing disease following treatment completion. TB-associated mortality and disability were determined by TB status and FEV1 percentage. Individuals surviving TB disease were assumed to face ongoing morbidity and mortality risks due to post-TB sequelae. The magnitude of these risks was based on the extent of residual long impairment as measured by FEV1 percentage. As compared to the earlier application of the model [21], this analysis adopted higher values for the FEV1 distribution for individuals with TB, reflecting a milder distribution of TB disease compared to individuals detected through passive case detection. Key model parameters are provided in **Table A** in **S1 Appendix**, and the model was programmed in R and C++. Additional details are reported in Can et al., 2025 [21].” [lines 144-175]

37. The costs to patients are identical whether they are receiving TB treatment or not (appendix table 2). Is this correct? It is somewhat surprising.

**Response**: These values were estimated from available data from the Malawi National TB and Leprosy Elimination Programme, which did not support disaggregation by TB disease phase. Over the course of a disease episode, individuals with an extended period of untreated TB would experience higher costs compared to those diagnosed earlier through ACF.

38. Is the cost of active case finding estimated on a per person basis or on a per TB episode diagnosed basis? From the way it is written, it appears the latter, but this value seems particularly susceptible to underlying prevalence and would vary greatly among the clusters screened.

**Response**: Thank you for identifying this. These costs were per-person, not per TB episode. We have revised the text to correct this.

39. Was uncertainty integrated into the diagnostic algorithm shown in appendix table 3?

**Response**: Yes, uncertainty was integrated into the diagnostic algorithm. The associated parameters, their values/assumptions (with uncertainty ranges), and prior distributions are now reported in **Table C** in **S1 Appendix.**

**Table C**: Parameters for the ACF diagnostic algorithm.

| **Parameter** | **Value/Assumption (95% interval)** | **Prior distribution** | **Source** |
| --- | --- | --- | --- |
| Sensitivity, symptom screen  (any cough) | 42.0% (31.5, 52.5) | Beta (35.0, 48.4) | [35] |
| Sensitivity, chest X-ray  (any abnormality) | 94.0% (91.0, 97.0) | Beta (223, 14.2) | [35] |
| Sensitivity, Xpert MTB/RIF | 84.7% (78.6, 89.9) | Beta (130, 23.5) | [36,37] |
| Specificity, symptom screen  (any cough) | 94.0% (92.5, 95.5) | Beta (902, 57.5) | [35] |
| Specificity, chest X-ray  (any abnormality) | 89.0% (86.3, 91.8) | Beta (440, 54.4) | [35] |
| Specificity, Xpert MTB/RIF | 99.80% (99.75, 99.85) | Beta (30521, 61.2) | [36,37] |
| Pre-treatment LTFU | 5.0% (2.5,7.5) | Beta (14.4, 273) | NTLEP, personal communication, 2025 |
