# Supplementary material for: Potential health impacts and costs of active case finding guided by Mycobacterium tuberculosis immunoreactivity survey results in Blantyre, Malawi: A mathematical modeling study
Source: PLoS Med. 2026 Oct 7;23(10):e1005272. doi: 10.1371/journal.pmed.1005272 (PMC13657404; doi:10.1371/journal.pmed.1005272)
Supplement: Supplementary file 4 [file pmed.1005272.s004.docx]

**Potential health impacts and costs of active case finding guided by Mycobacterium tuberculosis immunoreactivity survey results in Blantyre, Malawi (PMEDICINE-D-25-03673R2)**

**Response to reviewers**
**Reviewer #1:**

1. **On comment #2:** Evaluating the impact of a single round of screening when theoretically (and in previous empirical studies) the benefits are only likely to be evident after multiple rounds of screening is NOT a proof of concept. Furthermore, since the risk of transmission (and hence secondary cases) is related to community prevalence, there is a good reason to expect that, for the incidence outcome, there will not be diminishing returns, but in fact, accentuated returns from multiple rounds of screening. The concept of diminishing returns assumes no benefits from preventing secondary and subsequent cases due to transmission. While there is a substantial cost in collecting empirical data from multiple rounds of screenings, there is no such cost from doing this in a modelling study. Hence, if the authors truly want to test the proof of concept, they should present the evaluation of multiple rounds of screening - with an endpoint being prevalence reduced to a specified level.

**Response**: We thank the reviewer for this comment. The modelling framework developed for this study was designed to investigate the impact of a single round of ACF, and expanding the analysis to consider multiple rounds would require substantial expansion of both model and manuscript. Single-round ACF is an approach that is considered and implemented in many programmatic contexts, and so we believe the manuscript is valuable even if not expanded to address the impact of multiple-round ACF.

We agree with the reviewer that the Discussion should not draw conclusions about diminishing returns of multiple-round ACF, given that this was not investigated in the analysis. In response, we have revised the manuscript to remove language suggesting that repeated rounds of screening would necessarily yield diminishing returns. We additionally expanded the Discussion section to acknowledge that repeated rounds of ACF may generate further population-level benefits through reductions in downstream transmission. Specifically, we rephrased the relevant sentence as:

*“Fifth, in this study we examined the impact and cost-effectiveness of a single round of ACF. However, in some settings ACF will be implemented as repeated rounds, with each round generating additional health benefits by identifying further people with TB. For ACF implemented as multiple rounds, the long-term health effects will depend on changes in disease prevalence over time and resulting dynamic effects on TB transmission [49]. We did not explicitly model repeated ACF rounds, and the incremental costs and benefits of such strategies may differ from those presented here.”*

2. **On comment #3:** Good to see this additional analysis. However, it seems to be based on the assumption that ACF will reduce transmission by 50% compared with PCF. Not sure where this estimate comes from. It should be noted that most cases detected by ACF may never have been detected by PCF, since they are asymptomatic. While some may have become symptomatic (and hence be available for PCF) others will not have and will remain infectious until self-healing. In these instances, transmission risk will have been reduced by 100%. In a minority of cases, the individual may have been subsequently diagnosed with PCF, in which case there would be a partial reduction in transmission risk.

**Response**: We thank the reviewer for this comment. This assumption of 50% transmission reduction is based on the observation that, for an epidemic in equilibrium, a cross-sectional intervention like ACF will identify individuals half-way through their duration of diagnosable disease, on expectation (i.e., those with recently incident disease will be balanced by those with long-standing disease). This will be true for individuals who would have otherwise been diagnosed and treated via PCF, as well as for those who would have otherwise not been detected via PCF (and therefore died with TB disease, or self-cured). For this reason, we disagree with the reviewer’s suggestion that transmission risk will be reduced by 100% for individuals who would have subsequently self-healed.

However, we agree that there is substantial uncertainty regarding the magnitude of transmission reduction resulting from ACF, due to uncertainty in how transmission is concentrated within the disease episode, epidemiological context, and the impact of any short-term changes in passive detection. We also agree that these considerations only apply to the index case identified through ACF. For secondary TB cases, prevention of that case would avert 100% of the onward transmission associated with that case.

In response to the Reviewer’s comment, we have expanded our examination of transmission effects and epidemiological context. Specifically, we have included sensitivity analyses for two additional scenarios:

1. A scenario with greater transmission reductions for each TB case identified through ACF, assuming that ACF reduces transmission by 75% (with uncertainty bounds spanning 50–100%);
2. A scenario examining an epidemiological context with a substantially higher baseline TB prevalence (452 per 100,000, based on estimates from Malawi’s national tuberculosis prevalence survey (2013-2014) as reported by WHO).

Moreover, we have adjusted our main analysis to consider all future secondary TB cases (as compared to the last revision in which the main analysis only considered the first generation of secondary cases).

Across these analyses, the health impact and cost-effectiveness of ACF is greater than estimates in the previous revision (edits described below). However, the findings regarding the primary comparison of the study (targeted vs. untargeted ACF) remained unchanged: targeted ACF was consistently more cost-effective than untargeted ACF, and greater ARTI predictive power was associated with larger health gains and more favorable ICERs.

The Methods, Results, and Discussion sections were revised accordingly, and the additional sensitivity analyses are now presented in **Table M** of the Supplementary Appendix. The methods edits are shown below. As the change to the main analysis (to consider all future secondary TB cases) changes values throughout the Results section, we have not quoted those changes below.

*“For individuals with TB identified through ACF we assumed transmission would be reduced by 50% (with an uncertainty interval from 25% to 75%) as compared to the PCF scenario. This was based on the rationale that a single cross-sectional ACF round will identify individuals approximately half-way through their infectious period, on expectation. We used the reproductive number to calculate the geometric series of future TB cases averted by ACF.”*

*“We considered costs and health benefits associated with all future secondary TB cases averted by ACF. In sensitivity analyses, we recalculated costs and health consequences i) assuming ACF would reduce transmission among detected TB cases by 75% (vs. 50% in the main analysis), ii) using a higher TB prevalence estimate (452 per 100,000, based on data for Blantyre extracted from Malawi’s 2013-2014 national TB prevalence survey [cite], vs. 215 per 100,000 in the main analysis), and iii) excluding transmission benefits entirely.”*

Cite: Malawi Tuberculosis Prevalence survey, technical report: Ministry of Health, National TB Control Programme; 2013-2014.

3. **On comment #5**: I would argue that having a focus on individuals with TB, rather the population at risk of TB, remains an underlying problem with the interpretation of this analysis. This approach is suitable for modelling noncommunicable diseases, but is not, in my opinion, suitable for modelling the impact of interventions on communicable diseases (particularly those with airborne, human-to-human transmission, such as TB, influenza and COVID-19). This problem is not solved by simply looking at two generations of cases (index cases and their secondary cases). It requires a model that looks at population effects over time.

**Response**: Thank you. In response to the Reviewer’s comment, we have revised the analysis so that the main results now reflect the health and economic consequences for index cases as well as all subsequent generations of transmission (rather than being limited to the first-generation of secondary cases). As such, the analysis now considers all individuals potentially affected by ACF (either being included in the ACF intervention, or through developing TB in the future). While this approach does not produce the population-average results requested by the reviewer (as would be required to report population-level changes in the force of infection over time, for example), it is consistent with the approach required for cost-effectiveness, which (by design) is only concerned with incremental costs and health benefits for individuals in some way affected by the intervention.

4. **On comments #4, #6, and #7**: Responses are helpful.

**Response**: Thank you.

5. **On comment #39**: Table C shows specificity of "any cough" being 94%. The implication is that only 6% of people in the general population without TB acknowledge any cough at any given time. This seems fairly implausible. Furthermore, Table 6, in the cited source WHO report gives a specificity value of 0.88 (88%).

**Response**: We appreciate the Reviewer catching this error. The reference to “any cough” in Table C was a labeling oversight; this should have been labeled “prolonged cough”. With this correction, the values are consistent with the evidence in the WHO report that the reviewer cites. The underlying parameter values and results remain unchanged.
