# Supplementary material for: Potential health impacts and costs of active case finding guided by Mycobacterium tuberculosis immunoreactivity survey results in Blantyre, Malawi: A mathematical modeling study
Source: PLoS Med. 2026 Oct 7;23(10):e1005272. doi: 10.1371/journal.pmed.1005272 (PMC13657404; doi:10.1371/journal.pmed.1005272)
Supplement: Supplementary file 5 [file pmed.1005272.s005.docx]

**Potential health impacts and costs of active case finding guided by Mycobacterium tuberculosis immunoreactivity survey results in Blantyre, Malawi (PMEDICINE-D-25-03673R3)**

**Response to reviewers**

**Reviewer #1:**

Thanks to the authors for their considered and detailed responses to my previous comments. My only additional comment would be that the conclusion in the Abstract that the observed "cost-effectiveness ratios exceeded available cost-effectiveness thresholds for Malawi" only applies to the main analysis. The ICER is highly sensitive to the two alternative assumptions: higher impact of transmission and higher baseline prevalence. I wonder if this should be acknowledged in the Abstract.

**Response**: Thank you for this suggestion. We agree that the statement regarding cost-effectiveness thresholds applies only to the main analysis. We have revised the Abstract (Methods and Findings) to clarify this point by adding the following text:

*“In the main analysis, these cost-effectiveness ratios exceeded available cost-effectiveness thresholds for Malawi, although cost-effectiveness improved under assumptions of higher TB prevalence or greater transmission reduction. The findings are based on mathematical modeling and depend on assumptions regarding the relationship between ARTI and TB prevalence, which remains uncertain.”*
