# Supplementary material for: Potential health impacts and costs of active case finding guided by Mycobacterium tuberculosis immunoreactivity survey results in Blantyre, Malawi: A mathematical modeling study
Source: PLoS Med. 2026 Oct 7;23(10):e1005272. doi: 10.1371/journal.pmed.1005272 (PMC13657404; doi:10.1371/journal.pmed.1005272)
Supplement: Peer review history

# Editor report: Decision Letter 0

**DOI:** 10.1371/journal.pmed.1005272.r001

**Authors:** Heather Van Epps

21 Oct 2025

Dear Dr Kim,

Thank you for submitting your manuscript entitled "Potential health impacts and costs of active case finding guided by Mycobacterium tuberculosis immunoreactivity survey results in Blantyre, Malawi" for consideration by PLOS Medicine.

Your manuscript has now been evaluated by the PLOS Medicine editorial staff, and I am writing to let you know that we would like to send your submission out for external peer review.

However, before we can send your manuscript to reviewers, we need you to complete your submission by providing the metadata that is required for full assessment. To this end, please login to Editorial Manager where you will find the paper in the 'Submissions Needing Revisions' folder on your homepage. Please click 'Revise Submission' from the Action Links and complete all additional questions in the submission questionnaire.

For clinical studies, please upload a copy of your trial study protocol as a supporting information file. The study protocol should be the version submitted for approval to the institutional review board or ethics committee, should include any amendments to the study protocol, as well as the date of their approval by the institutional review or ethics committee. Please also detail any deviations from the study protocol in the Methods section of your manuscript. The editors will consider the protocol and study conduct prior to a final decision for external review.

Please re-submit your manuscript within two working days, i.e. by October 23rd.

Login to Editorial Manager here: https://www.editorialmanager.com/pmedicine

Once your full submission is complete, your paper will undergo a series of checks in preparation for peer review. Once your manuscript has passed all checks it will be sent out for review.

Feel free to email us at plosmedicine@plos.org if you have any queries relating to your submission.

Kind regards,

Heather Van Epps, PhD

Consulting Editor

PLOS Medicine

# Peer review: Decision Letter 1

**DOI:** 10.1371/journal.pmed.1005272.r002

**Authors:** Andreia Cunha

2 Dec 2025

Dear Dr Kim,

Sincere apologies for the delay in getting back to you with a decision, which was due to challenges in securing all the necessary advice. Many thanks for submitting your manuscript "Potential health impacts and costs of active case finding guided by Mycobacterium tuberculosis immunoreactivity survey results in Blantyre, Malawi" (PMEDICINE-D-25-03673R1) to PLOS Medicine. The paper has been reviewed by subject experts and a statistician; their comments are included below and can also be accessed here: [LINK]

As you will see, the reviewers find your work of great interest and they raise several important points that we must ask you to resolve in full. After discussing the paper with the editorial team and an academic editor with relevant expertise, I'm pleased to invite you to revise the paper in response to the reviewers' comments. We plan to send the revised paper to some or all of the original reviewers, and we cannot provide any guarantees at this stage regarding publication.

In addition to these revisions, you may need to complete some formatting changes, which you will receive in a follow up email. A member of our team will be in touch with a set of requests shortly. If you do not receive a separate email within a few days, please assume that checks have been completed, and no additional changes are required.

When you upload your revision, please include a point-by-point response that addresses all of the reviewer and editorial points, indicating the changes made in the manuscript and either an excerpt of the revised text or the location (eg: page and line number) where each change can be found. Please also be sure to check the general editorial comments at the end of this letter and include these in your point-by-point response. When you resubmit your paper, please include a clean version of the paper as the main article file and a version with changes tracked as a marked-up manuscript. It may also be helpful to check the guidelines for revised papers at http://journals.plos.org/plosmedicine/s/revising-your-manuscript for any that apply to your paper.

We ask that you submit your revision by Jan 05 2026 11:59PM. However, if this deadline is not feasible, please contact me by email, and we can discuss a suitable alternative.

Don't hesitate to contact me directly with any questions (acunha@plos.org).

Best regards,

Andreia

Andreia Cunha, PhD

Senior editor

PLOS Medicine

acunha@plos.org

-----------------------------------------------------------

Comments from the academic editor:

The points raised by Reviewers 1, 4 and 5 are of particular importance to address.

-----------------------------------------------------------

Comments from the reviewers:

Reviewer #1: The study is clearly and thoroughly analysed and presented. However, I have a number of questions that do not appear to be covered in the description of the intervention and measurement of outcomes (effectiveness).

1.What is the justification for limiting the ACF to one round of screening? Both empirical and modelling studies of ACF have shown that benefits are cumulative and repeated screening is required to demonstrate effectiveness on prevalence, transmission and incidence, and hence on health outcomes (mortality and DALYs).

2.Does the model incorporate the effect of the intervention on transmission and subsequent incidence (which is additional to the benefit for the individuals who are diagnosed by the intervention). Without including these compounding benefits, the effectiveness is substantially underestimated and hence ICERs are substantially over-estimated.

3.Related to the above, what is the time horizon for measuring the outcomes?

4.Results are cited as " improve life expectancy by …. for individuals with TB disease". This is not a relevant outcome. The relevant outcome is improved life expectancy (and reduction in DALYs) for the population as a whole and this includes the effective on incidence of TB prevented over a long time horizon due to reduced transmission (due to reduced prevalence).

5.What are the assumptions about participation rates in screening and rates of successful transition for TB diagnosis to completing TB treatment (in all three arms)?

6.My other comment would be that the cost of screening using the proposed algorithm may be over-estimated based on newer developments. I realise this may not need to be estimated but it should be acknowledged that ICER would be lower if the cost of screening could be lowered, based on evolution of new tools, without losing effectiveness.

Reviewer #2: In their manuscript "Potential health impacts and costs of active case finding guided by Mycobacterium tuberculosis immunoreactivity survey results in Blantyre, Malawi", Kim et al. present a mathematical model assessing the effect and cost-effectiveness of a tuberculosis (TB) active case finding (ACF) intervention. In the model, the geographical implementation of the ACF intervention is guided by TB immunoreactivity survey results in children <5 years, serving as a surrogate parameter for the annual risk of TB infection. Overall, I find the study to be very well conducted, with methods presented clearly, valuable results, and a sound introduction and discussion. Below, I offer a few comments the authors might want to consider to further improve the manuscript.

Methods:

-On page 4, the authors describe the setting the model is parameterized to. Given that Blantyre, Malawi is a setting with a high TB and HIV burden, the generalisability of the model's results to other epidemiological settings is limited. Thus, perhaps as a sensitivity analysis, I would suggest to assess how the model's results change in settings with a lower TB and HIV burden.

-On page 5, I found a detailed description of the ACF intervention, but only very limited information about the passive case-finding (PCF) only scenario. I would suggest to add a brief description of the PCF scenario, including the tool to diagnose TB, linkage to treatment, and whether treatment outcomes differ for individuals detected through PCF versus ACF.

-On page 6, 2022 USD are defined as the currency used in the model. Is there a reason 2022 USD are used instead of 2025 USD? I would suggest that the authors either state this reason or update costs to 2025 USD.

-On page 7, a 5% pre-treatment loss to follow-up is described as part of the ACF intervention. However, I could not find any description of loss to follow-up due to inability to expectorate sufficient sputum for Xpert testing, even though the proportion of individuals not able to produce sputum can be substantial (see e.g., https://doi.org/10.1101/2025.11.02.25339326). It would be very helpful if the authors could clarify whether the possibility of insufficient sputum expectoration is considered in the model. In case the possibility of insufficient sputum expectoration is not yet included in the model, I would to incorporate it.

Results:

-On page 10, in the last sentence of the first paragraph, I would suggest to state the exact average decrease in life-expectancy through unnecessary TB treatment instead of stating 'limited but non-negligible impact'.

-On page 21, table 2, please specify why the bottom six ICERs are marked in grey - in the appendix, only clusters where targeted ACF is dominated are marked in grey.

Discussion:

-On page 13/14, in the paragraph discussing the availability of Mtb immunoreactivity survey data, it would be helpful if the authors could include a few examples where such data for children <5 years is readily available.

-On page 14, in the last paragraph, the authors argue that their findings of ACF potentially mitigating excess TB-associated mortality contrasts the findings of the TREATS study, which did not find a relevant epidemiological effect of a combined community-based TB/HIV testing and care intervention. They mention the exclusion of dynamic transmission effects in their model as one of the potential reasons for this difference. To me, this reads as if the difference in results would have been smaller had dynamic transmission effects been included in the present model. Is this what the authors meant to say? Shouldn't the inclusion of dynamic transmission effects lead to an even larger benefit of ACF, thus further increasing in the difference in results of the present model and the results of the TREATS study?

Reviewer #3: Thank you to the authors for submitting this excellent paper, I very much enjoyed reading it. It is on a clearly important topic, very well written and has a nice balanced discussion of the results. I have a few minor comments that I think the authors may want to look at before publication can be considered.

- I think Appendix Figure 1 needs to be moved into the main text

- We need much more information about the Markov model. This is should be in the main text rather than the appendices. Can't assume that the reader will have read the Can et al (2025) paper

- I think we need the information from Appendix Table 1 in the main text, either as an actual table or described in the text

- I think we need a little more information regarding the key costs in the main text too. Not all the costs from Appendix Table 2, but some of the key ones described in the text to get a feel for the type of level of costs we're dealing with

- I think you need to be a little clearer regarding the different intervention scenarios in the model. In the 'Intervention Scenarios' section you talk about PCF Only, Untargeted ACF and Targeted ACF, whereas the results only report ACF vs no ACF. It's a little confusing.

- I'm a little confused where the 48% figure came when assessing the the implications of geographical targeting. Is just because it was roughly half and fitted the clusters? One sentence regarding this would be helpful

- In the discussion you state that unlike the TREATS model you didn't include dynamic transmission effects in your model. I was wondering if the authors could comment on why they didn't include this in their model, how much of a disadvantage this is and how it may impact the results of their study

- Is there a better way of presenting the results in Table 1, Appendix Table 5 and Appendix Table 6? It looks really odd and a bit messy having different numbers of decimal places in different rows of the table

Reviewer #4: Kim and collagues presnnt their analysis of the impact of ACf vs PCF in Malawi, for a strategy of targeted intervention guided to specific districts in Blantyre using survey data from Mtb immunoreactivity under 5 years olds.

Major Comments

1. Lack of detail regarding the underlying model structure and inputs

The analysis relies heavily on a Markov microsimulation model described as being "developed by Can et al. (2025)" with only a brief reference stating "The model incorporated transitions between health states, including undiagnosed TB, diagnosis and treatment, post-TB sequelae, and death" (page 6, Methods). This creates a significant transparency issue as readers must consult an external publication to understand fundamental aspects of the analysis, like the complete model structure and health states, transition probabilities between states, natural history assumptions, how HIV status affects transitions, age stratification and demographic processes

In light of this, the Methods section should be restructured to first provide a clear, self-contained description of the model architecture before explaining how it was applied to this specific question. Consider adding a diagram showing the complete model structure with all health states and transitions, a table of key transition parameters, clear statement that this analysis applies a previously published and validated model to a new question (ARTI-guided targeting) and an

explicit description of which aspects are novel to this study versus which are from the prior work.

2. Post-TB sequelae modeling

The manuscript mentions that the model includes "post-TB sequelae" as a health state and notes these "are increasingly recognized as major contributors to TB disease burden" (page 14, Discussion). The model appears to account for post-TB morbidity through YLDs and ongoing productivity losses (Appendix Table 2). However, critical details are missing, namely:

What specific post-TB sequelae are included (respiratory impairment, cardiovascular complications, etc.)?

What proportion of treated/untreated TB cases progress to sequelae?

How do sequelae severity and duration differ by HIV status?

What disability weights are applied?

How do post-TB sequelae affect mortality risk?

Given that post-TB sequelae appear to influence the cost-effectiveness estimates (through ongoing costs and DALYs), these assumptions require transparent reporting.

Perhaps add a dedicated subsection or table describing the post-TB sequelae model component, including transition probabilities to sequelae states, disability weights, mortality risks, and costs associated with this state. If these details are only in Can et al. (2025), they should be summarized here given their importance to the current analysis.

3. MTB immunoreactivity surveys as a targeting tool

While the novelty of using Mtb immunoreactivity surveys in children <5 years for ACF targeting is central to this work, there is insufficient critical discussion of this approach's strengths and limitations. The manuscript briefly mentions that "immunoreactivity surveys are considerably less intensive than TB prevalence surveys" (page 14) and that the survey used convenience sampling from primary care, but several important issues receive inadequate attention, like feasibility and acceptability of testing young children; selection bias from convenience sampling (children attending clinics may not represent community burden); Uncertainty in ARTI estimates, particularly in clusters with zero positives (acknowledged briefly but not discussed in depth); The assumption that infection risk in children <5 reflects recent transmission and correlates with prevalent disease (which is central but examined only through sensitivity analysis)

The manuscript acknowledges in limitations that "the strength of this relationship [between ARTI and TB prevalence] may vary" but doesn't adequately discuss the implications of the convenience sampling approach or situate this method among alternatives.

Additional Comments

Minor point on model description: The Methods would benefit from explicitly stating upfront (perhaps in the first paragraph of "Modelling approach") that the analysis applies a previously developed and validated TB natural history model to assess a novel intervention targeting strategy. Currently, this only becomes clear midway through the Methods section.

These comments aim to help the authors improve transparency and strengthen the manuscript's contribution. The work addresses an important question, but clearer presentation of the underlying model and more critical engagement with the surveillance approach would substantially enhance its value to the field.

Reviewer #5: Sun and colleagues use mathematical models to evaluate the effectiveness and cost-effectiveness of active TB case finding in specific areas of Blantyre, Malawi, either in a universal manner, or targeted to higher risk areas based on estimated annual risk of TB infection. They conclude that when compared to passive case finding, any active approach prevents TB and DALYs, but that a targeted approach is more efficient (cost-effective) than a universal approach. The authors present an intriguing way to think about how to target case finding, highlighting the use of TB infection surveys as a more inexpensive alternative to identifying higher risk areas than activities like prevalence surveys.

MAJOR

-The authors do not include the costs of the initial TB infection prevalence survey that was used to inform the targeting of case finding. While in this specific circumstance the survey was already performed, this will not always be the case. The authors acknowledge this will add cost, but this is a potentially important cost and would be important to include as it would be necessary for implementation elsewhere. Costs of this survey could be annuitized over the expected timeframe from when such a survey would need to be repeated, as if indeed active case finding was guided by such surveys, their impact on epidemiology would need to be monitored so they could be continuously re-targeted.

-The authors do not consider the impacts of their intervention on transmission. This is another acknowledged limitation, but an important one, particularly in a high-burden setting with an epidemic driven by HIV. In some of the clusters, estimated annual risk of infection was as high as 8%, suggesting high-levels of ongoing transmission. I do not think the inclusion of transmission would necessarily reduce the estimated ICERs to local willingness-to-pay thresholds, but I do think it may have an important impact, particularly in these estimated-to-be high transmission areas.

MINOR

-Do the authors have TB incidence among each of the clusters included in the annual risk of infection analysis? Some clusters had no infection detected during the survey. Knowing the incidence data for these clusters in relation to the estimated annual risk of infection would provide the reader some ability to understand the face validity of the annual risk of infection estimates (i.e., are they reasonable, given the convenience sampling approach used).

-I understand many model details are published elsewhere, but given no limitations on the length of the supplement, it would be helpful to have the complete list of modeling parameters and approaches included for ease of reference. Given the aim of the study referenced (Can et al) is to look at progressive lung damage and implications of different treatment delays on this outcome, necessary modifications to Blantyre (a setting not included in the original model) and the specific questions evaluated here, should be explained. Can the authors also supply sources for demographic parameters used (e.g., I was surprised to see 75% of the population male).

-The costs to patients are identical whether they are receiving TB treatment or not (appendix table 2). Is this correct? It is somewhat surprising.

-Is the cost of active case finding estimated on a per person basis or on a per TB episode diagnosed basis? From the way it is written, it appears the latter, but this value seems particularly susceptible to underlying prevalence and would vary greatly among the clusters screened.

-Was uncertainty integrated into the diagnostic algorithm shown in appendix table 3?

Any attachments provided with reviews can be seen via the following link: [LINK]

--------------------------------------------------------- ---

General editorial requests:

(Note: not all will apply to your paper, but please check each item carefully)

*We ask every co-author listed on the manuscript to fill in a contributing author statement, making sure to declare all competing interests. If any of the co-authors have not filled in the statement, we will remind them to do so when the paper is revised. If all statements are not completed in a timely fashion this could hold up the re-review process. If new competing interests are declared later in the revision process, this may also hold up the submission. Should there be a problem getting one of your co-authors to fill in a statement we will be in contact. Please do not add or remove authors without first discussing this with the handling editor. You can see our competing interests policy here: http://journals.plos.org/plosmedicine/s/competing-interests.

*Please upload any figures associated with your paper as individual TIF or EPS files with 300dpi resolution at resubmission; please read our figure guidelines for more information on our requirements: http://journals.plos.org/plosmedicine/s/figures. While revising your submission, we strongly recommend that you use PLOS's NAAS tool (https://ngplosjournals.pagemajik.ai/artanalysis) to test your figure files. NAAS can convert your figure files to the TIFF file type and meet basic requirements (such as print size, resolution), or provide you with a report on issues that do not meet our requirements and that NAAS cannot fix.

After uploading your figures to PLOS's NAAS tool - https://ngplosjournals.pagemajik.ai/artanalysis, NAAS will process the files provided and display the results in the "Uploaded Files" section of the page as the processing is complete.

If the uploaded figures meet our requirements (or NAAS is able to fix the files to meet our requirements), the figure will be marked as "fixed" above. If NAAS is unable to fix the files, a red "failed" label will appear above.

When NAAS has confirmed that the figure files meet our requirements, please download the file via the download option, and include these NAAS processed figure files when submitting your revised manuscript.

*Please ensure that the paper adheres to the PLOS Data Availability Policy (see http://journals.plos.org/plosmedicine/s/data-availability), which requires that all data underlying the study's findings be provided in a repository or as Supporting Information. For data residing with a third party, authors are required to provide instructions with contact information (web or email address) for obtaining the data. Please note that a study author cannot be the contact person for the data. PLOS journals do not allow statements supported by "data not shown" or "unpublished results." For such statements, authors must provide supporting data or cite public sources that include it.

*We expect all researchers with submissions to PLOS in which author-generated code underpins the findings in the manuscript to make all author-generated code available without restrictions upon publication of the work. In cases where code is central to the manuscript, we may require the code to be made available as a condition of publication. Authors are responsible for ensuring that the code is reusable and well documented. Please make any custom code available, either as part of your data deposition or as a supplementary file. Please add a sentence to your data availability statement regarding any code used in the study, e.g. "The code used in the analysis is available from Github [URL] and archived in Zenodo [DOI link]" Please review our guidelines at https://journals.plos.org/plosmedicine/s/materials-software-and-code-sharing and ensure that your code is shared in a way that follows best practice and facilitates reproducibility and reuse. Because Github depositions can be readily changed or deleted, we encourage you to make a permanent DOI'd copy (e.g. in Zenodo) and provide the URL.

*At this stage, we ask that you include a short, non-technical Author Summary of your research to make findings accessible to a wide audience that includes both scientists and non-scientists. The Author Summary should immediately follow the Abstract in your revised manuscript. This text is subject to editorial change and should be distinct from the scientific abstract. Ideally each sub-heading should contain 2-3 single sentence, concise bullet points containing the most salient points from your study. In the final bullet point of 'What Do These Findings Mean?', please include the main limitations of the study in non-technical language. Please see our author guidelines for more information: https://journals.plos.org/plosmedicine/s/revising-your-manuscript#loc-author-summary.

*Please express the main results with 95% CIs as well as p values. When reporting p values please report as p<0.001 and where higher as the exact p value p=0.002, for example. Throughout, suggest reporting statistical information as follows to improve clarity for the reader "22% (95% CI [13%,28%]; p</=)". Please be sure to define all numerical values at first use.

*Please include page numbers and line numbers in the manuscript file. Use continuous line numbers (do not restart the numbering on each page).

*Please cite the reference numbers in square brackets. Citations should precede punctuation.

FIGURES AND TABLES

*Please provide titles and legends for all figures and tables (including those in Supporting Information files).

*Please define all abbreviations used in each figure/table (including those in Supporting Information files).

*Please consider avoiding the use of red and green in order to make your figure more accessible to those with color blindness.

SUPPLEMENTARY MATERIAL

*Please note that supplementary material will be posted as supplied by the authors. Therefore, please amend it according to the relevant comments outlined here.

*Please cite your Supporting Information as outlined here: https://journals.plos.org/plosmedicine/s/supporting-information

REFERENCES

*PLOS uses the numbered citation (citation-sequence) method and first six authors, et al.

*Please ensure that journal name abbreviations match those found in the National Center for Biotechnology Information (NCBI) databases (http://www.ncbi.nlm.nih.gov/nlmcatalog/journals), and are appropriately formatted and capitalised.

*Where website addresses are cited, please include the complete URL and specify the date of access (e.g. [accessed: 12/06/2023]).

*Please also see https://journals.plos.org/plosmedicine/s/submission-guidelines#loc-references for further details on reference formatting.

MODELLING STUDIES

The following list is derived from Geoffrey P Garnett, Simon Cousens, Timothy B Hallett, Richard Steketee, Neff Walker. Mathematical models in the evaluation of health programmes. (2011) Lancet DOI:10.1016/S0140-6736(10)61505-X:

*If pertinent, please provide a diagram that shows the model structure, including how the natural history of the disease is represented, the process and determinants of disease acquisition, and how the putative intervention could affect the system.

*Please provide a complete list of model parameters, including clear and precise descriptions of the meaning of each parameter, together with the values or ranges for each, with justification or the primary source cited and important caveats about the use of these values noted.

*Please provide a clear statement about how the model was fitted to the data, including goodness-of-fit measure, the numerical algorithm used, which parameter varied, constraints imposed on parameter values, and starting conditions.

*For uncertainty analyses, please state the sources of uncertainties quantified and not quantified [can include parameter, data, and model structure].

*Please provide sensitivity analyses to identify which parameter values are most important in the model. Uncertainty estimates seek to derive a range of credible results on the basis of an exploration of the range of reasonable parameter values. The choice of method should be presented and justified.

*Please discuss the scientific rationale for the choice of model structure and identify points where this choice could influence conclusions drawn. Please also describe the strength of the scientific basis underlying the key model assumptions.

*For studies that develop a prediction model or evaluate its performance, please ensure that the study is reported according to the TRIPOD statement (https://www.equator-network.org/reporting-guidelines/tripod-statement) and include the completed checklist as Supporting Information. Please add the following statement, or similar, to the Methods: "This study is reported as per the Transparent Reporting of a Multivariable Prediction Model for Individual Prognosis Or Diagnosis (TRIPOD) statement (S1 Checklist)." For studies using machine learning, please use the TRIPOD-AI checklist. When completing the checklist, please use section and paragraph numbers, rather than page numbers.

HEALTH ECONOMICS / COST-EFFECTIVENESS STUDIES

*Please ensure that the study is reported according to the CHEERS guideline (available from: https://www.equator-network.org/reporting-guidelines/cheers) and include the completed checklist as Supporting Information. Please add the following statement, or similar, to the Methods: "This study is reported as per the Strengthening the Consolidated Health Economic Evaluation Reporting Standards 2022 (CHEERS 2022) Statement (S1 Checklist)." When completing the checklist, please use section and paragraph numbers, rather than page numbers.

# Author comment: Author response to Decision Letter 2

**DOI:** 10.1371/journal.pmed.1005272.r003

2 Mar 2026

Attachment Submitted filename: Response-to-Reviewer-PlosMed-Malawi-SunKim-Feb28, 2026.docx

# Peer review: Decision Letter 2

**DOI:** 10.1371/journal.pmed.1005272.r004

**Authors:** Andreia Cunha

27 Apr 2026

Dear Dr Kim,

Sincere apologies for the delay in getting back to you with a decision, which was due to challenges in securing all the necessary advice. Many thanks for submitting your manuscript "Potential health impacts and costs of active case finding guided by Mycobacterium tuberculosis immunoreactivity survey results in Blantyre, Malawi" (PMEDICINE-D-25-03673R2) to PLOS Medicine. The paper has been reviewed by subject experts and a statistician; their comments are included below and can also be accessed here: [LINK]

As you will see, Reviewer 1 still maintains some important concerns that we must ask you to resolve in full. After discussing the paper with the editorial team and an academic editor with relevant expertise, I'm pleased to invite you to revise the paper in response to the reviewers' comments. We plan to send the revised paper to some or all of the original reviewers, and we cannot provide any guarantees at this stage regarding publication.

In addition to these revisions, you may need to complete some formatting changes, which you will receive in a follow up email. A member of our team will be in touch with a set of requests shortly. If you do not receive a separate email within a few days, please assume that checks have been completed, and no additional changes are required.

When you upload your revision, please include a point-by-point response that addresses all of the reviewer and editorial points, indicating the changes made in the manuscript and either an excerpt of the revised text or the location (eg: page and line number) where each change can be found. Please also be sure to check the general editorial comments at the end of this letter and include these in your point-by-point response. When you resubmit your paper, please include a clean version of the paper as the main article file and a version with changes tracked as a marked-up manuscript. It may also be helpful to check the guidelines for revised papers at http://journals.plos.org/plosmedicine/s/revising-your-manuscript for any that apply to your paper.

We ask that you submit your revision by May 18 2026 11:59PM. However, if this deadline is not feasible, please contact me by email, and we can discuss a suitable alternative.

Don't hesitate to contact me directly with any questions (acunha@plos.org).

Best regards,

Andreia

Andreia Cunha, PhD

Senior editor

PLOS Medicine

acunha@plos.org

-----------------------------------------------------------

Comments from the reviewers:

Reviewer #1: Comment #2.

Evaluating the impact of a single round of screening when theoretically (and in previous empirical studies) the benefits are only likely to be evident after multiple rounds of screening is NOT a proof of concept. Furthermore, since the risk of transmission (and hence secondary cases) is related to community prevalence, there is a good reason to expect that, for the incidence outcome, there will not be diminishing returns, but in fact, accentuated returns from multiple rounds of screening. The concept of diminishing returns assumes no benefits from preventing secondary and subsequent cases due to transmission.

While there is a substantial cost in collecting empirical data from multiple rounds of screenings, there is no such cost from doing this in a modelling study. Hence, if the authors truly want to test the proof of concept, they should present the evaluation of multiple rounds of screening - with an endpoint being prevalence reduced to a specified level.

Comment #3.

Good to see this additional analysis. However, it seems to be based on the assumption that ACF will reduce transmission by 50% compared with PCF. Not sure where this estimate comes from. It should be noted that most cases detected by ACF may never have been detected by PCF, since they are asymptomatic. While some may have become symptomatic (and hence be available for PCF) others will not have and will remain infectious until self-healing. In these instances, transmission risk will have been reduced by 100%. In a minority of cases, the individual may have been subsequently diagnosed with PCF, in which case there would be a partial reduction in transmission risk.

Comment #5

I would argue that having a focus on individuals with TB, rather the population at risk of TB, remains an underlying problem with the interpretation of this analysis. This approach is suitable for modelling noncommunicable diseases, but is not, in my opinion, suitable for modelling the impact of interventions on communicable diseases (particularly those with airborne, human-to-human transmission, such as TB, influenza and COVID-19). This problem is not solved by simply looking at two generations of cases (index cases and their secondary cases). It requires a model that looks at population effects over time.

Comments #4, #6 and #7.

Responses are helpful.

Comment #39. Table C shows specificity of "any cough" being 94%. The implication is that only 6% of people in the general population without TB acknowledge any cough at any given time. This seems fairly implausible. Furthermore, Table 6, in the cited source WHO report gives a specificity value of 0.88 (88%).

Guy Marks

Reviewer #2: I would like to thank the authors for their detailed consideration of the reviewers' suggestions. I don't have further comments.

Reviewer #3: The authors have responded to all of my comments in a satisfactory manner. I'm happy to approve this one.

Reviewer #4: The authors have addressed my main concerns and the manuscript could be accepted

Reviewer #5: The authors have addressed the most critical comments of the reviewers adequately and amended where required.

Any attachments provided with reviews can be seen via the following link: [LINK]

--------------------------------------------------------- ---

General editorial requests:

(Note: not all will apply to your paper, but please check each item carefully)

*We ask every co-author listed on the manuscript to fill in a contributing author statement, making sure to declare all competing interests. If any of the co-authors have not filled in the statement, we will remind them to do so when the paper is revised. If all statements are not completed in a timely fashion this could hold up the re-review process. If new competing interests are declared later in the revision process, this may also hold up the submission. Should there be a problem getting one of your co-authors to fill in a statement we will be in contact. Please do not add or remove authors without first discussing this with the handling editor. You can see our competing interests policy here: http://journals.plos.org/plosmedicine/s/competing-interests.

*Please upload any figures associated with your paper as individual TIF or EPS files with 300dpi resolution at resubmission; please read our figure guidelines for more information on our requirements: http://journals.plos.org/plosmedicine/s/figures. While revising your submission, we strongly recommend that you use PLOS's NAAS tool (https://ngplosjournals.pagemajik.ai/artanalysis) to test your figure files. NAAS can convert your figure files to the TIFF file type and meet basic requirements (such as print size, resolution), or provide you with a report on issues that do not meet our requirements and that NAAS cannot fix.

After uploading your figures to PLOS's NAAS tool - https://ngplosjournals.pagemajik.ai/artanalysis, NAAS will process the files provided and display the results in the "Uploaded Files" section of the page as the processing is complete.

If the uploaded figures meet our requirements (or NAAS is able to fix the files to meet our requirements), the figure will be marked as "fixed" above. If NAAS is unable to fix the files, a red "failed" label will appear above.

When NAAS has confirmed that the figure files meet our requirements, please download the file via the download option, and include these NAAS processed figure files when submitting your revised manuscript.

*Please ensure that the paper adheres to the PLOS Data Availability Policy (see http://journals.plos.org/plosmedicine/s/data-availability), which requires that all data underlying the study's findings be provided in a repository or as Supporting Information. For data residing with a third party, authors are required to provide instructions with contact information (web or email address) for obtaining the data. Please note that a study author cannot be the contact person for the data. PLOS journals do not allow statements supported by "data not shown" or "unpublished results." For such statements, authors must provide supporting data or cite public sources that include it.

*We expect all researchers with submissions to PLOS in which author-generated code underpins the findings in the manuscript to make all author-generated code available without restrictions upon publication of the work. In cases where code is central to the manuscript, we may require the code to be made available as a condition of publication. Authors are responsible for ensuring that the code is reusable and well documented. Please make any custom code available, either as part of your data deposition or as a supplementary file. Please add a sentence to your data availability statement regarding any code used in the study, e.g. "The code used in the analysis is available from Github [URL] and archived in Zenodo [DOI link]" Please review our guidelines at https://journals.plos.org/plosmedicine/s/materials-software-and-code-sharing and ensure that your code is shared in a way that follows best practice and facilitates reproducibility and reuse. Because Github depositions can be readily changed or deleted, we encourage you to make a permanent DOI'd copy (e.g. in Zenodo) and provide the URL.

FORMATTING - GENERAL

*Abstract: Please structure your abstract using the PLOS Medicine headings (Background, Methods and Findings, Conclusions). Please combine the Methods and Findings sections into one section.

*At this stage, we ask that you include a short, non-technical Author Summary of your research to make findings accessible to a wide audience that includes both scientists and non-scientists. The Author Summary should immediately follow the Abstract in your revised manuscript. This text is subject to editorial change and should be distinct from the scientific abstract. Ideally each sub-heading should contain 2-3 single sentence, concise bullet points containing the most salient points from your study. In the final bullet point of 'What Do These Findings Mean?', please include the main limitations of the study in non-technical language. Please see our author guidelines for more information: https://journals.plos.org/plosmedicine/s/revising-your-manuscript#loc-author-summary.

*Please express the main results with 95% CIs as well as p values. When reporting p values please report as p<0.001 and where higher as the exact p value p=0.002, for example. Throughout, suggest reporting statistical information as follows to improve clarity for the reader "22% (95% CI [13%,28%]; p</=)". Please be sure to define all numerical values at first use.

*Please include page numbers and line numbers in the manuscript file. Use continuous line numbers (do not restart the numbering on each page).

*Please cite the reference numbers in square brackets. Citations should precede punctuation.

FIGURES AND TABLES

*Please provide titles and legends for all figures and tables (including those in Supporting Information files).

*Please define all abbreviations used in each figure/table (including those in Supporting Information files).

*Please consider avoiding the use of red and green in order to make your figure more accessible to those with color blindness.

SUPPLEMENTARY MATERIAL

*Please note that supplementary material will be posted as supplied by the authors. Therefore, please amend it according to the relevant comments outlined here.

*Please cite your Supporting Information as outlined here: https://journals.plos.org/plosmedicine/s/supporting-information

REFERENCES

*PLOS uses the numbered citation (citation-sequence) method and first six authors, et al.

*Please ensure that journal name abbreviations match those found in the National Center for Biotechnology Information (NCBI) databases (http://www.ncbi.nlm.nih.gov/nlmcatalog/journals), and are appropriately formatted and capitalised.

*Where website addresses are cited, please include the complete URL and specify the date of access (e.g. [accessed: 12/06/2023]).

*Please also see https://journals.plos.org/plosmedicine/s/submission-guidelines#loc-references for further details on reference formatting.

OBSERVATIONAL STUDIES

*Abstract: Please include the study design, population and setting, number of participants, years during which the study took place (enrollment and follow up), length of follow up, and main outcome measures.

*Please ensure that the study is reported according to the STROBE (or appropriate STOBE extension) guideline (available from: https://www.equator-network.org/reporting-guidelines/strobe) and include the completed STROBE (or STROBE extension) checklist as Supporting Information. Please add the following statement, or similar, to the Methods: "This study is reported as per the Strengthening the Reporting of Observational Studies in Epidemiology (STROBE) guideline (S1 Checklist)." When completing the checklist, please use section and paragraph numbers, rather than page numbers.

*FOR POPULATION HEALTH/REGISTRY STUDIES: Please ensure that the study is reported according to the RECORD guideline (available from https://www.record-statement.org) and include the completed checklist as Supporting Information. Please add the following statement, or similar, to the Methods: "This study is reported as per the Reporting of Studies Conducted using Observational Routinely-Collected Data (RECORD) guideline (S1 Checklist)." When completing the checklist, please use section and paragraph numbers, rather than page numbers.

*FOR POPULATION HEALTH ESTIMATES: Please ensure that the study is reported according to the GATHER statement (available from https://www.equator-network.org/reporting-guidelines/gather-statement) and include the completed checklist as Supporting Information. Please add the following statement, or similar, to the Methods: "This study is reported as per the Guidelines for Accurate and Transparent Health Estimates Reporting (GATHER) statement (S1 Checklist)." When completing the checklist, please use section and paragraph numbers, rather than page numbers.

*FOR MEDIATION ANALYSES: We recommend that the study is reported according to the AGReMA statement (https://agrema-statement.org/#:~:text=AGReMA%20is%20an%20evidence%2D%20and,randomised%20trials%20and%20observational%20studies) and include the completed checklist as Supporting Information. Please add the following statement, or similar, to the Methods: "This study is reported as per the Guideline for Reporting Mediation Analyses (AGReMA) statement (S1 Checklist)." When completing the checklist, please use section and paragraph numbers, rather than page numbers.

*For all observational studies, in the manuscript text, please indicate: (1) the specific hypotheses you intended to test, (2) the analytical methods by which you planned to test them, (3) the analyses you actually performed, and (4) when reported analyses differ from those that were planned, transparent explanations for differences that affect the reliability of the study's results. If a reported analysis was performed based on an interesting but unanticipated pattern in the data, please be clear that the analysis was data driven.

*Please state in the Methods section whether the study had a prospective protocol or analysis plan. If a prospective analysis plan (from your funding proposal, IRB or other ethics committee submission, study protocol, or other planning document written before analyzing the data) was used in designing the study, please include the relevant document(s) with your revised manuscript as a Supporting Information file to be published alongside your study and cite it in the Methods section. A legend for this file should be included at the end of your manuscript. If no such document exists, please make sure that the Methods section transparently describes when analyses were planned, and when/why any data-driven changes to analyses took place. Changes in the analysis, including those made in response to peer review comments, should be identified as such in the Methods section of the paper, with rationale.

SURVEY-BASED STUDIES

*Please ensure that the study is reported according to the CROSS guideline (https://www.equator-network.org/reporting-guidelines/a-consensus-based-checklist-for-reporting-of-survey-studies-cross/) and include the completed CROSS checklist as Supporting Information. Please add the following statement, or similar, to the Methods: "This study is reported as per A Consensus-Based Checklist for Reporting of Survey Studies (CROSS) guideline (S1 Checklist)." When completing the checklist, please use section and paragraph numbers, rather than page numbers.

*Please report your survey response rates according to AAPOR recommendations (https://aapor.org/standards-and-ethics/best-practices/)

*Please define how the population surveyed was sampled.

*Please compare characteristics of respondents and nonrespondents if possible.

*If sequential waves of the survey were sent, please specify whether the characteristics of respondents changed over time or remained constant.

*Please include the survey response rate in the Abstract.

*Please include a copy of the survey in the supplementary files.

# Author comment: Author response to Decision Letter 3

**DOI:** 10.1371/journal.pmed.1005272.r005

1 Jun 2026

Attachment Submitted filename: Response-to-Reviewer-PLOS-Med-Malawi-SunKim-June1,2026.docx

# Peer review: Decision Letter 3

**DOI:** 10.1371/journal.pmed.1005272.r006

**Authors:** Andreia Cunha

20 Jul 2026

Dear Dr. Kim,

Thank you very much for re-submitting your manuscript "Potential health impacts and costs of active case finding guided by Mycobacterium tuberculosis immunoreactivity survey results in Blantyre, Malawi" (PMEDICINE-D-25-03673R3) for review by PLOS Medicine.

I have discussed the paper with my colleagues and the academic editor and it was also seen again by one of the original reviewers. I am pleased to say that provided the remaining editorial and production issues are dealt with we are planning to accept the paper for publication in the journal.

The remaining issues that need to be addressed are listed at the end of this email. Any accompanying reviewer attachments can be seen via the link below. Please take these into account before resubmitting your manuscript:

[LINK]

***Please note while forming your response, if your article is accepted, you may have the opportunity to make the peer review history publicly available. The record will include editor decision letters (with reviews) and your responses to reviewer comments. If eligible, we will contact you to opt in or out.***

In revising the manuscript for further consideration here, please ensure you address the specific points made by each reviewer and the editors. In your rebuttal letter you should indicate your response to the reviewers' and editors' comments and the changes you have made in the manuscript. Please submit a clean version of the paper as the main article file. A version with changes marked must also be uploaded as a marked up manuscript file.

Please also check the guidelines for revised papers at http://journals.plos.org/plosmedicine/s/revising-your-manuscript for any that apply to your paper. If you haven't already, we ask that you provide a short, non-technical Author Summary of your research to make findings accessible to a wide audience that includes both scientists and non-scientists. The Author Summary should immediately follow the Abstract in your revised manuscript. This text is subject to editorial change and should be distinct from the scientific abstract.

In addition to these revisions, you may need to complete some formatting changes, which you will receive in a follow up email. A member of our team will be in touch with a set of requests shortly. If you do not receive a separate email within a few days, please assume that checks have been completed, and no additional changes are required.

We expect to receive your revised manuscript within 1 week. Please email us (plosmedicine@plos.org) if you have any questions or concerns.

We ask every co-author listed on the manuscript to fill in a contributing author statement. If any of the co-authors have not filled in the statement, we will remind them to do so when the paper is revised. If all statements are not completed in a timely fashion this could hold up the re-review process. Should there be a problem getting one of your co-authors to fill in a statement we will be in contact. YOU MUST NOT ADD OR REMOVE AUTHORS UNLESS YOU HAVE ALERTED THE EDITOR HANDLING THE MANUSCRIPT TO THE CHANGE AND THEY SPECIFICALLY HAVE AGREED TO IT.

Please ensure that the paper adheres to the PLOS Data Availability Policy (see http://journals.plos.org/plosmedicine/s/data-availability), which requires that all data underlying the study's findings be provided in a repository or as Supporting Information. For data residing with a third party, authors are required to provide instructions with contact information for obtaining the data. PLOS journals do not allow statements supported by "data not shown" or "unpublished results." For such statements, authors must provide supporting data or cite public sources that include it.

To enhance the reproducibility of your results, we recommend that you deposit your laboratory protocols in protocols.io, where a protocol can be assigned its own identifier (DOI) such that it can be cited independently in the future. Additionally, PLOS ONE offers an option to publish peer-reviewed clinical study protocols. Read more information on sharing protocols at https://plos.org/protocols?utm_medium=editorial-email&utm_source=authorletters&utm_campaign=protocols

Please review your reference list to ensure that it is complete and correct. If you have cited papers that have been retracted, please include the rationale for doing so in the manuscript text, or remove these references and replace them with relevant current references. Any changes to the reference list should be mentioned in the rebuttal letter that accompanies your revised manuscript.

Please note, when your manuscript is accepted, an uncorrected proof of your manuscript will be published online ahead of the final version, unless you've already opted out via the online submission form. If, for any reason, you do not want an earlier version of your manuscript published online or are unsure if you have already indicated as such, please let the journal staff know immediately at plosmedicine@plos.org.

If you have any questions in the meantime, please contact me or the journal staff on plosmedicine@plos.org.

We look forward to receiving the revised manuscript by Jul 24 2026 11:59PM.

Sincerely,

Andreia Cunha, PhD

Senior Editor

PLOS Medicine

plosmedicine.org

------------------------------------------------------------

Requests from Editors:

GENERAL EDITORIAL REQUESTS

* At this stage, we ask that you include a short, non-technical Author Summary of your research to make findings accessible to a wide audience that includes both scientists and non-scientists. The Author Summary should immediately follow the Abstract in your revised manuscript. This text is subject to editorial change and should be distinct from the scientific abstract. Ideally each sub-heading should contain 2-3 single sentence, concise bullet points containing the most salient points from your study. In the final bullet point of ‘What Do These Findings Mean?’ Please include the main limitations of the study in non-technical language.

Please see our author guidelines for more information: https://journals.plos.org/plosmedicine/s/revising-your-manuscript#loc-author-summary."

* Please confirm that your title complies with PLOS Medicine's style. Your title must be nondeclarative and not a question. It should begin with main concept if possible. "Effect of" should be used only if causality can be inferred, i.e., for an RCT. Please place the study design ("A randomized controlled trial," "A retrospective study," "A modelling study," etc.) in the subtitle (ie, after a colon).

* Please confirm that your abstract complies with our requirements, including format (three sections: Background, Methods and Findings, and Conclusions) and providing all the information relevant to this study type https://journals.plos.org/plosmedicine/s/submission-guidelines#loc-abstract

* Please ensure that the Introduction ends with a clear description of the study question or hypothesis.

* Please ensure that all abbreviations are defined at first use throughout the text.

* Please confirm that all numbers presented in the abstract are present and identical to numbers presented in the main manuscript text.

GENERAL

* Please disclose any use of AI tools and technologies to the study or article content at the end of the Methods. Please specify the name(s) and version(s) of any tool(s) used and a description of their use, including how the AI-assisted or generated content was evaluated by authors, and a clear statement of which aspects of the study, article contents, data, or supporting files were affected/generated by AI tool usage. Please also include a statement specifying who retains responsibility for the final manuscript. See our guidelines for more information: https://journals.plos.org/plosmedicine/s/ethical-publishing-practice#loc-artificial-intelligence-tools-and-technologies.

* Please review your text for claims of novelty or primacy (e.g. 'for the first time') and remove this language. In addition, please check that any use of statistical terms (such as trend or significant) are supported by the data, and if not please remove them.

* Statistical reporting: Please revise throughout the manuscript, including tables and figures.

- Please report statistical information as follows to improve clarity for the reader ""22% (95% CI [13,28]; p</=)"".

- Please separate upper and lower bounds with commas instead of hyphens as the latter can be confused with reporting of negative values.

- Please repeat statistical definitions (HR, CI etc.) for each set of parentheses."

* In the last sentence of the Abstract Methods and Findings section, please describe the main limitation(s) of the study's methodology.

* In the abstract, please include the important dependent variables that are adjusted for in the analyses.

FUNDING STATEMENT

* The funding statement should include: specific grant numbers, initials of authors who received each award, URLs to sponsors’ websites. Also, please state whether any sponsors or funders (other than the named authors) played any role in study design, data collection and analysis, the decision to publish, or preparation of the manuscript. If they had no role in the research, include this sentence: “The funders had no role in study design, data collection and analysis, decision to publish, or preparation of the manuscript.”

COMPETING INTERESTS STATEMENT

* All authors must declare their relevant competing interests per the PLOS policy, which can be seen here: https://journals.plos.org/plosmedicine/s/competing-interests For authors with ties to industry, please indicate whether any of the interests has a financial stake in the results of the current study.

*Please amend your competing interests statement. Funding information should go to the relevant section and include URLs.

* Please add this statement to the manuscript's Competing Interests: "PM is an Academic Editor on PLOS Medicine's editorial board. EC was a Guest Editor on PLOS Medicine."

DATA AVAILABILITY

* PLOS defines the “minimal data set” to consist of the data set used to reach the conclusions drawn in the manuscript with related metadata and methods, and any additional data required to replicate the reported study findings in their entirety. Authors do not need to submit their entire data set, or the raw data collected during an investigation. Please submit the following data:

The values behind the means, standard deviations and other measures reported;

The values used to build graphs;

The points extracted from images for analysis.

* The Data Availability Statement (DAS) requires revision. For each data source used in your study:

a) If the data are freely or publicly available, note this and state the location of the data: within the paper, in Supporting Information files, or in a public repository (include the DOI or accession number).

b) If the data are owned by a third party but freely available upon request, please note this and state the institutional owner of the data set and contact information for data requests (insitutional web or email address). Note that a study author cannot be the contact for the data.

c) If the data are not freely available, please describe briefly the ethical, legal, or contractual restriction that prevents you from sharing it. Please also include an appropriate institutional contact (insitutional web or email address) for inquiries (again, this cannot be a study author).

* For studies in which a novel model is central to the manuscript's findings, as is the case here, authors are responsible for providing the source code needed to replicate the study's findings in a repository (such as GitHub, SourceForge or Bitbucket) or a cloud computing service (such as Code Ocean). Please include a citable digital object identifier (DOI) or other persistent identifier of the code (such as through linkage with Zenodo). Protection of authors’ intellectual property will not be cause for exception. Please explain in the manuscript’s Data Availability Statement how readers can access the shared code. Please include the statement on code availability in the data availability statement. Please see guidance here:https://journals.plos.org/plosmedicine/s/code-sharing-guidance#loc-data-availability-statement-examples

ETHICS AND CONSENT

* Please add a statement explaining why ethical approval and informed consent were not required, since human clinical data was used in the study.

FIGURES

* Please consider avoiding the use of red and green in order to make your figure more accessible

* Please confirm that the appropriate usage rights apply to the use of this map. Please see our guidelines for map images: https://journals.plos.org/plosmedicine/s/figures#loc-maps

* Please provide the unadjusted comparisons as well as the adjusted comparisons in all relevant Tables

* Please specify the variables controlled for in all relevant Tables

* Please provide titles and legends for all figures and tables (including those in Supporting Information files). Please define all acronyms used in each figure or table in its corresponding legend.

* Please ensure that where relevant figures include 95% CIs.

* When a p value is given, please specify the statistical test used to determine it in the legend.

INCLUSIVITY IN GLOBAL RESEARCH

PLOS has an 'Inclusivity in Global Research' policy which aims to promote collaboration and inclusivity in global health research. You are required to complete PLOS’ questionnaire on inclusivity in global research and submit it with your revised paper. The policy and questionnaire can be found at https://journals.plos.org/plosone/s/best-practices-in-research-reporting.

MODELING STUDIES

For more details on the requests below please see Geoffrey P Garnett, Simon Cousens, Timothy B Hallett, Richard Steketee, Neff Walker. Mathematical models in the evaluation of health programmes. (2011) Lancet DOI:10.1016/S0140-6736(10)61505-X.

* Please provide a diagram that shows the model structure, including how the disease natural history is represented, the process and determinants of disease acquisition, and how the putative intervention could affect the system.

* Please provide a complete list of model parameters, including clear and precise descriptions of the meaning of each parameter, together with the values or ranges for each, with justification or the primary source cited, and important caveats about the use of these values noted.

* Please provide a clear statement about how the model was fitted to the data including where relevant goodness-of-fit measure, the numerical algorithm used, which parameter varied, constraints imposed on parameter values, and starting conditions.

* For uncertainty analyses, please state the sources of uncertainties quantified and not quantified this can include parameter, data, and model structure.

* Please provide sensitivity analyses to identify which parameter values are most important in the model. Uncertainty estimates seek to derive a range of credible results on the basis of an exploration of the range of reasonable parameter values. The choice of method should be presented and justified.

* Please discuss the scientific rationale for this choice of model structure and identify points where this choice could influence conclusions drawn. Please also describe the strength of the scientific basis underlying the key model assumptions.

Comments from Reviewers:

Reviewer #1: Thanks to the authors for their considered and detailed responses to my previous comments.

My only additional comment would be that the conclusion in the Abstract that the observed "cost-effectiveness ratios exceeded available cost-effectiveness thresholds for Malawi" only applies to the main analysis. The ICER is highly sensitive to the two alternative assumptions: higher impact of transmission and higher baseline prevalence. I wonder if this should be acknowledged in the Abstract.

Guy B. Marks

Any attachments provided with reviews can be seen via the following link:

[LINK]

# Author comment: Author response to Decision Letter 4

**DOI:** 10.1371/journal.pmed.1005272.r007

14 Sep 2026

Attachment Submitted filename: Response-to-Reviewer-PlosMed-Malawi-R3.docx

# Editor report: Decision Letter 4

**DOI:** 10.1371/journal.pmed.1005272.r008

**Authors:** Andreia Cunha

16 Sep 2026

Dear Dr Kim,

On behalf of my colleagues and the Academic Editor, Dr Alice Zwerling, I am pleased to inform you that we have agreed to publish your manuscript "Potential health impacts and costs of active case finding guided by Mycobacterium tuberculosis immunoreactivity survey results in Blantyre, Malawi: A mathematical modelling study" (PMEDICINE-D-25-03673R4) in PLOS Medicine.

Before your manuscript can be formally accepted you will need to complete some formatting changes, which you will also receive in a follow up email.

- Please make more explicit in the following sentences of the Abstract and Au Summary that these are the limitations of the study:

'The findings are based on mathematical modeling and depend on assumptions regarding the relationship between ARTI and TB prevalence, which remains uncertain.'

'Our findings are based on mathematical modeling and depend on assumptions regarding the relationship between ARTI and TB prevalence, transmission dynamics, and intervention effectiveness. Further empirical and implementation studies are needed to better characterize these relationships and evaluate the effectiveness of ARTI-guided targeted active case finding in real-world settings.'

- In the Abstract, please change the section heading 'Conclusion' to 'Conclusions'.

- Please revise the Funding statement to read as follows, since there is no need to mention open access licensing:

'HMR was funded by Wellcome (225482/Z/22/Z). PM was funded by Wellcome (304666/Z/23/Z, https://wellcome.org/research-funding/funding-portfolio/funded-grants/advancing

epidemiological-methods-surveillance) and an NIHR Global Health Research Professorship (NIHR304311, https://www.nihr.ac.uk/career-development/research career-funding-programmes/professorships-and-senior-investigators/global-health research-professorship/global-health-research-professors). The views expressed are those of the author(s) and not necessarily those of the NIHR or the Department of Health and Social Care. TC was funded by NIAID (R01AI147854, https://www.niaid.nih.gov/). The content is solely the responsibility of the authors and does not necessarily represent the official views of the National Institutes of Health. This work was funded by the UK aid from the UK government [to MDP, ELC, and PM; “Leaving no-one behind: transforming gendered pathways to health for TB; (2018/S 196-443482, https://devtracker.fcdo.gov.uk/programme/GB-CHC-222655

LIGHT/summary]; however, the views expressed do not necessarily reflect the UK government’s official policies. The funders had no role in study design, data collection and analysis, decision to publish, or preparation of the manuscript.'

- In the data availability statement, please include a citable digital object identifier (DOI) or other persistent identifier of the code (such as through linkage with Zenodo)

- Please clarify in the ethics statement if the data reused in the study was de-identified and analysed anonymously, and if this was the reason ethics approval and informed consent were not required.

Please be aware that it may take several days for you to receive this email; during this time no action is required by you. Once you have received these formatting requests, please note that your manuscript will not be scheduled for publication until you have made the required changes.

In the meantime, please log into Editorial Manager at http://www.editorialmanager.com/pmedicine/, click the "Update My Information" link at the top of the page, and update your user information to ensure an efficient production process.

PRESS

We frequently collaborate with press offices. If your institution or institutions have a press office, please notify them about your upcoming paper at this point, to enable them to help maximise its impact. If the press office is planning to promote your findings, we would be grateful if they could coordinate with medicinepress@plos.org. If you have not yet opted out of the early version process, we ask that you notify us immediately of any press plans so that we may do so on your behalf.

We also ask that you take this opportunity to read our Embargo Policy regarding the discussion, promotion and media coverage of work that is yet to be published by PLOS. As your manuscript is not yet published, it is bound by the conditions of our Embargo Policy. Please be aware that this policy is in place both to ensure that any press coverage of your article is fully substantiated and to provide a direct link between such coverage and the published work. For full details of our Embargo Policy, please visit http://www.plos.org/about/media-inquiries/embargo-policy/.

To enhance the reproducibility of your results, we recommend that you deposit your laboratory protocols in protocols.io, where a protocol can be assigned its own identifier (DOI) such that it can be cited independently in the future. Additionally, PLOS ONE offers an option to publish peer-reviewed clinical study protocols. Read more information on sharing protocols at https://plos.org/protocols?utm_medium=editorial-email&utm_source=authorletters&utm_campaign=protocols

Thank you again for submitting to PLOS Medicine. We look forward to publishing your paper.

Sincerely,

Andreia Cunha, PhD

Senior Editor

PLOS Medicine
